# Supplementary material for: Co-option of a non-retroviral endogenous viral element in planthoppers
Source: Nat Commun. 2023 Nov 9;14:7264. doi: 10.1038/s41467-023-43186-2 (PMC10636211; doi:10.1038/s41467-023-43186-2)
Supplement: Supplementary file 1 — Supplementary Information [file 41467_2023_43186_MOESM1_ESM.pdf]

Supplementary Information for

Co-option of a non-retroviral endogenous viral element in planthoppers

Hai-Jian Huang[1,2], Yi-Yuan Li[1,2], Zhuang-Xin Ye[1,2,3], Li-Li Li[1,2], Qing-Ling Hu[1,2], Yu-Juan He[1,2], Yu-Hua Qi[1,2], Yan Zhang[1,2], Ting Li[1,2], Gang Lu[1,2], Qian-Zhuo Mao[1,2], Ji-Chong Zhuo[1,2], Jia-Bao Lu[1,2], Zhong-Tian Xu[1,2], Zong-Tao Sun[1,2], Fei Yan[1,2], Jian-Ping Chen[1,2,3*], Chuan-Xi Zhang[1,2*], Jun-Min Li[1,2*]

[1] State Key Laboratory for Managing Biotic and Chemical Threats to the Quality and Safety of Agro-products, Institute of Plant Virology, Ningbo University, Ningbo 315211, China

[2] Key Laboratory of Biotechnology in Plant Protection of Ministry of Agriculture and Zhejiang Province, Institute of Plant Virology, Ningbo University, Ningbo 315211, China

[3] College of Forestry, Nanjing Forestry University, Nanjing 210037, China

* Corresponding authors:
Jian-Ping Chen, jianpingchen@nbu.edu.cn
Chuan-Xi Zhang, chxzhang@zju.edu.cn
Jun-Min Li, lijunmin@nbu.edu.cn

Supplementary Figures: 1-13

Supplementary Tables: 1-6

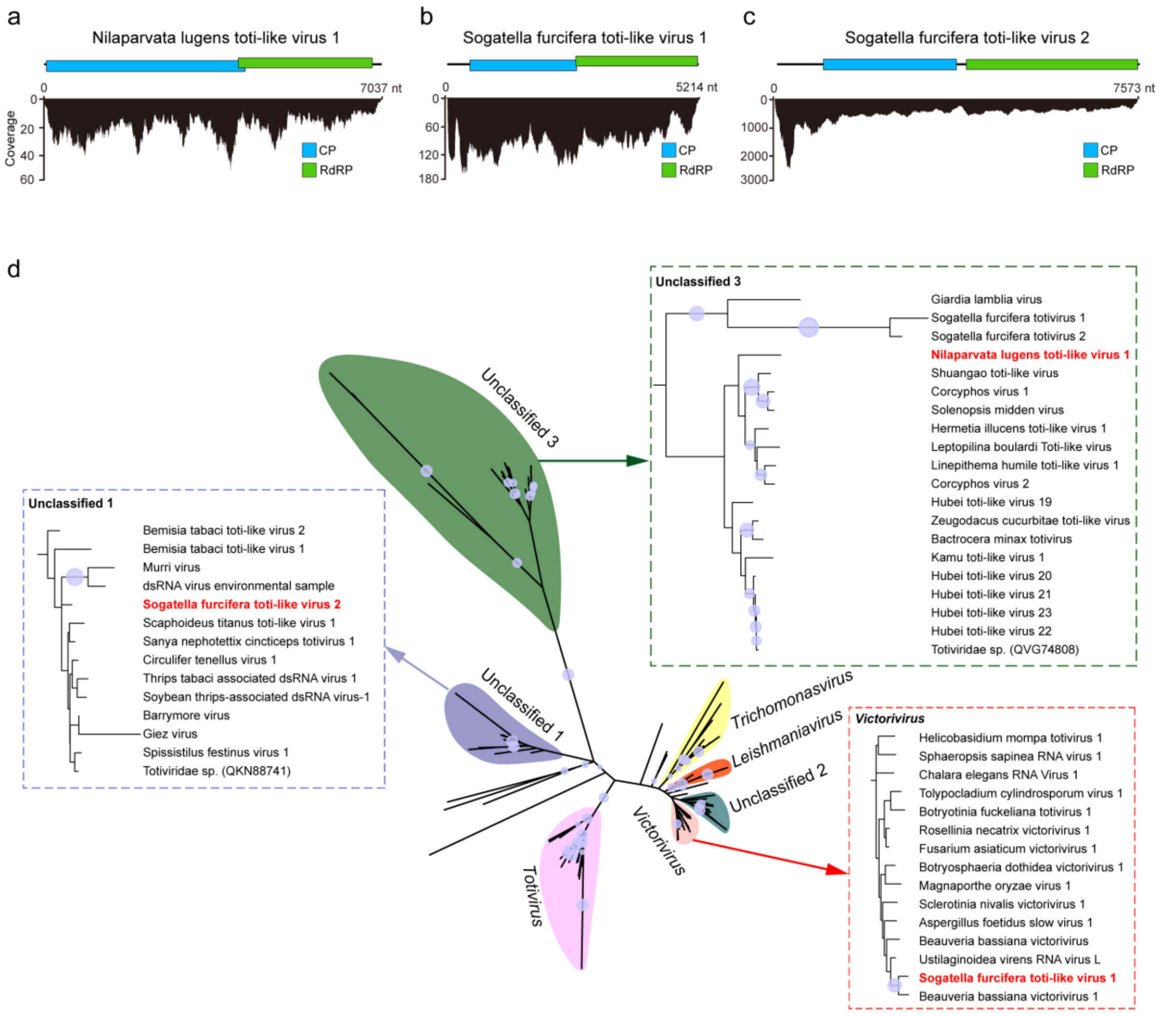

Supplemental Fig. 1: Genome structures and phylogeny of toti-like viruses identified in *Nilaparvata lugens* and *Sogatella furcifera*. Genome organization and read coverage of (a) Nilaparvata lugens toti-like virus 1 (NlToLV1), (b) Sogatella furcifera toti-like virus 1 (SfToLV1), and (c) Sogatella furcifera toti-like virus 2 (SfToLV2). (d) Maximum likelihood phylogenetic tree based on the RNA-dependent RNA polymerase of NlToLV1, SfToLV1, SfToLV2, and other toti/toti-like viruses. The toti-like viruses identified in this study are denoted in bold red font. Nodes with bootstrap values >50% are marked with solid blue circles, and the larger circles indicate higher bootstrap values. Source data are provided as a Source Data file.

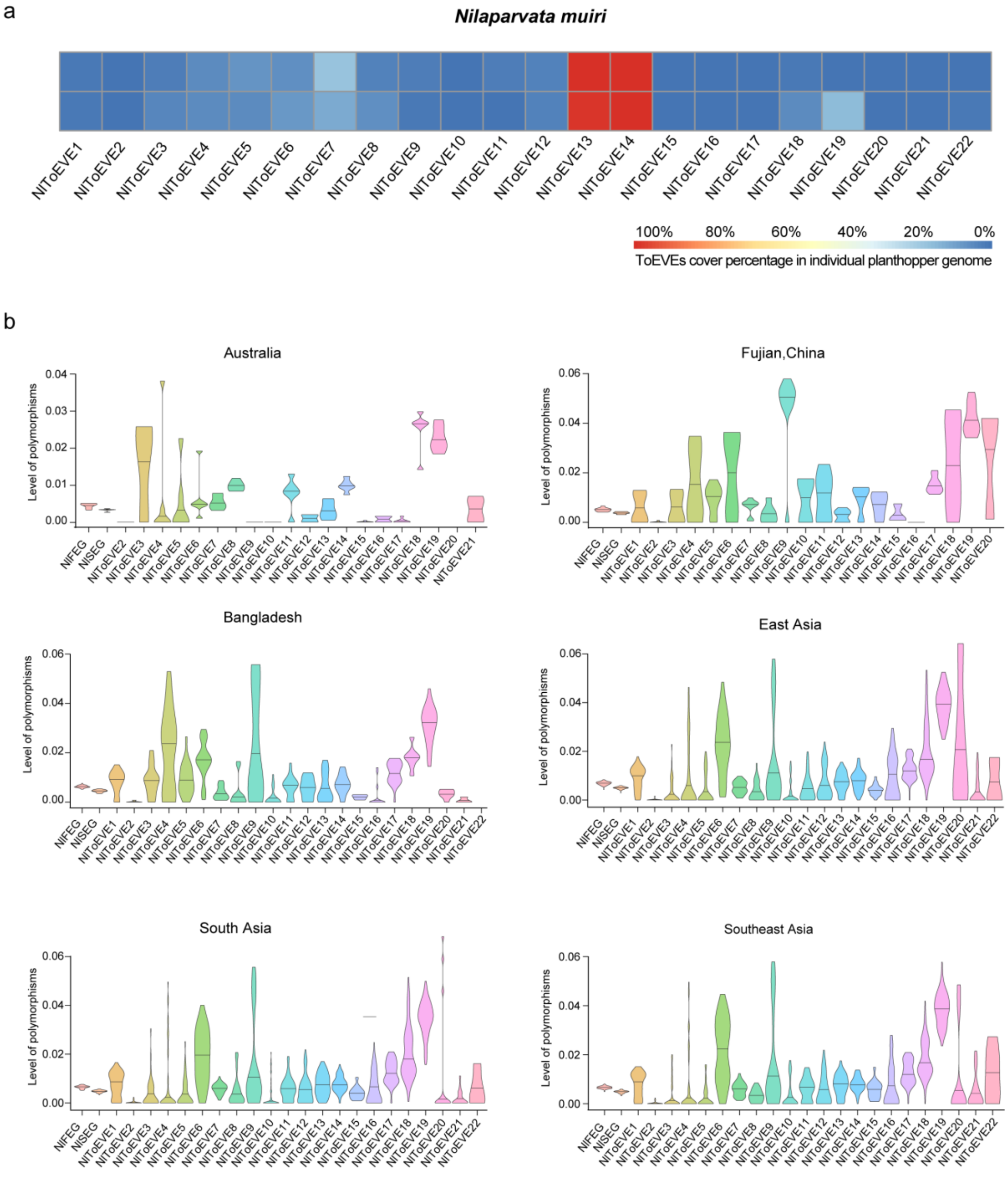

Supplemental Fig. 2: Distribution and polymorphism level of NlToEVEs in planthoppers. (a) NlToEVE prevalence (cover percentage) in two *Nilaparvata muiri* individuals. (b) Estimated polymorphism level for NlToEVEs in the six populations of *N. lugens*. Bars in violin plots correspond to the medians. n=6, 9, 25, 74, 72, and 70 individuals in Australia, Fujian, Bangladesh, East Asia, South Asia, and Southeast Asia populations, respectively. Source data are provided as a Source Data file.

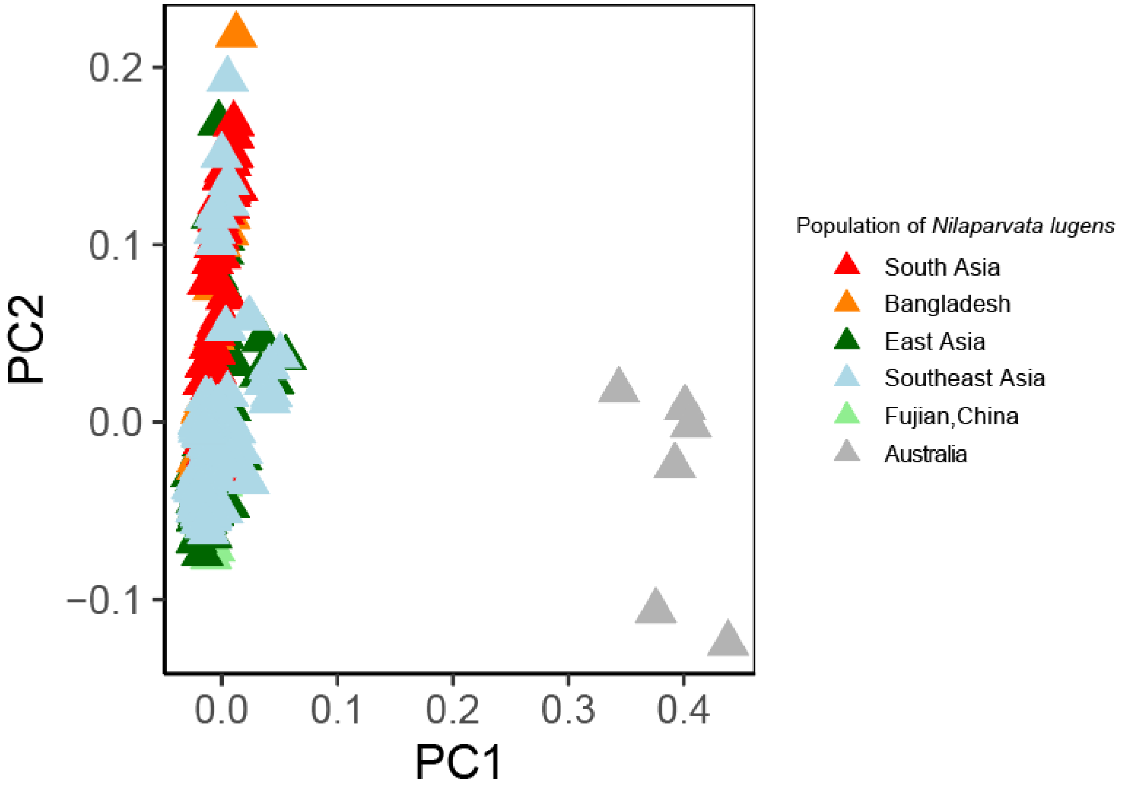

Supplemental Fig. 3: Principal component analysis (PCA) of the relative abundance and prevalence of NlToEVEs in different populations of *N. lugens*. Source data are provided as a Source Data file.

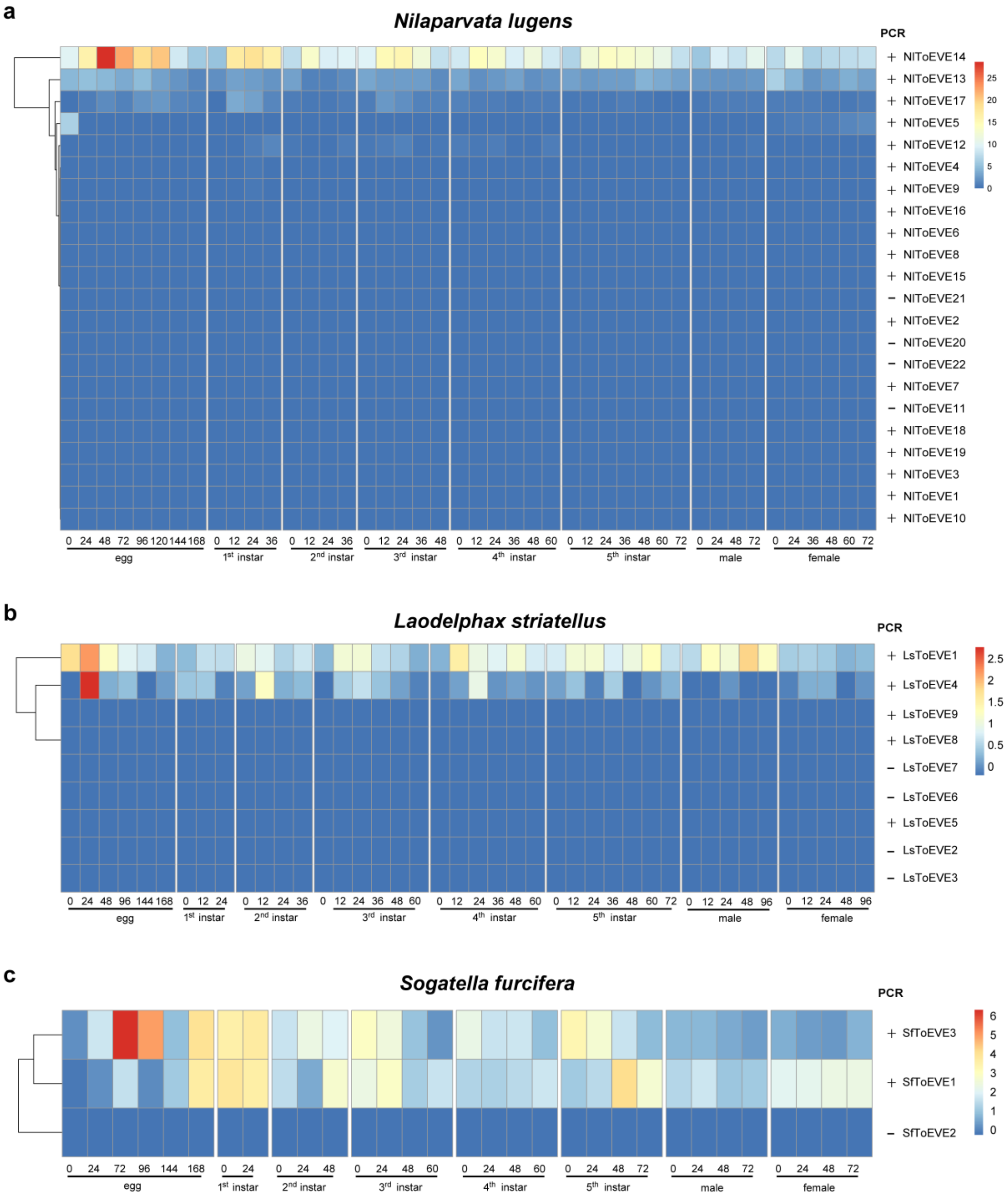

Supplemental Fig. 4: Heatmap representing the abundance of transcript reads derived from ToEVEs of *Nilaparvata lugens* (a), *Laodelphax striatellus* (b), and *Sogatella furcifera* (c) across various time points of different planthopper development stages. "+" indicates that the presence of the ToEVE transcript was confirmed by RT-PCR, while "-" shows the absence of the corresponding ToEVE transcript. Source data are provided as a Source Data file.

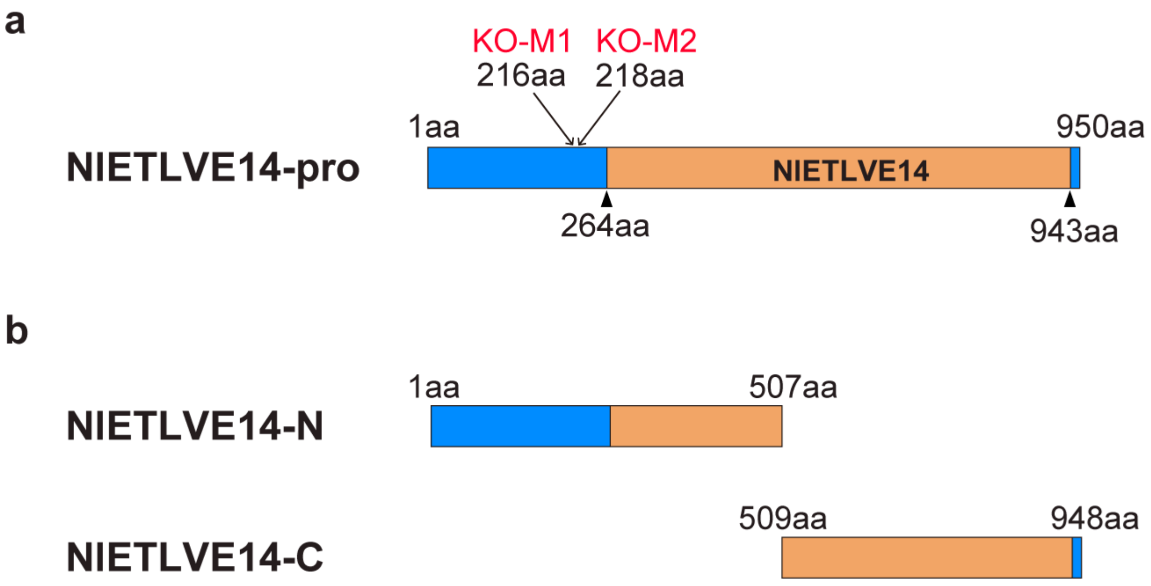

Supplemental Fig. 5: A schematic diagram of NlETLVE14 and the deletion mutants. (a) The predicated NlETLVE14 protein (NlETLVE14-pro) contained 950 amino acids (aa). The coat protein of Sogatella furcifera toti-like virus 2 exhibited homologous to NlETLVE14-pro from 264 aa to 926 aa (orange). Two mutant strains, KO-M1 and KO-M2, were obtained with frameshift mutation occurred after 216 aa and 218 aa, respectively. (b) The deletion mutants of NlETLVE14-pro. The N-terminal mutant of NlToEVE14 (NlToEVE14-N) contains sequences from 1 aa to 507 aa, while the C-terminal mutant of NlToEVE14 (NlToEVE14-C) contains sequences from 509 aa to 948 aa.

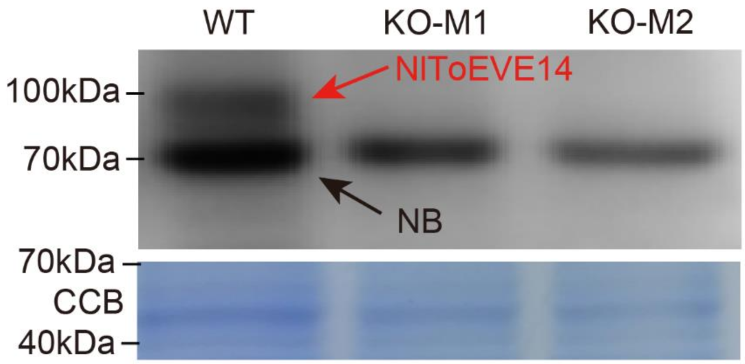

Supplemental Fig. 6: Detection of NlToEVE14 protein in wild-type (WT) and NlToEVE14 knockout mutants. The protein level of NlToEVE14 was detected in the WT and two NlToEVE14 knockout mutants (KO-M1 and KO-M2) using Western blotting. The band with the expected size of NlToEVE14 is indicated with a red arrow, whereas the non-specific band (NB) is marked with a black arrow. Coomassie brilliant blue (CBB) staining was conducted to visualize the amount of sample loading. Experiments were repeated three times with similar results. Source data are provided as a Source Data file.

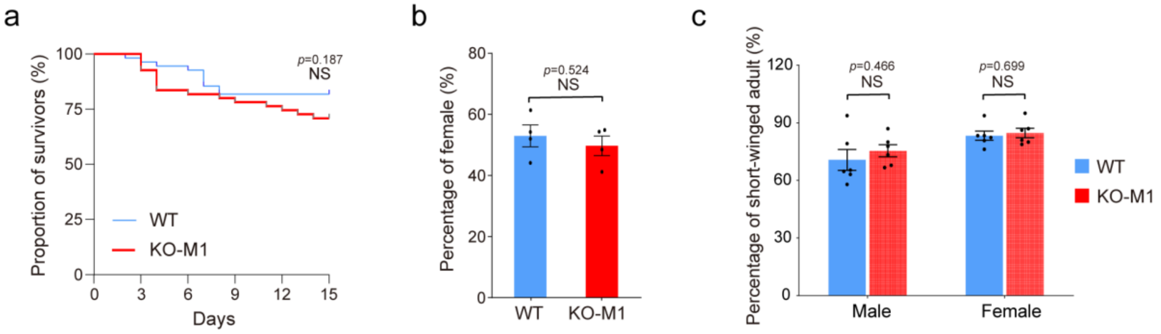

Supplemental Fig. 7: Effects of NlToEVE14 knockout on the survival rate of nymphs (a), the percentage of females (b), and the percentage of short-winged morphs (c) in comparisons between the KO-M1 and WT strains. Abbreviations, KO-M1: NlToEVE14 homozygous mutant strain 1 (4 bp deletion); WT: Wild-type population. For survival analysis in (a), n= 55 individuals in both WT and KO-M1; for sex analysis in (b), n= 4 independent biological replicates; for wing analysis in (c), n= 6 independent biological replicates. Data in (b) and (c) are presented as mean values ±SEM. *P*-value in (a) was determined by log-rank test, while *P*-values in (b) and (c) were determined by two-tailed unpaired Student's *t* test. NS, not significant. Source data are provided as a Source Data file.

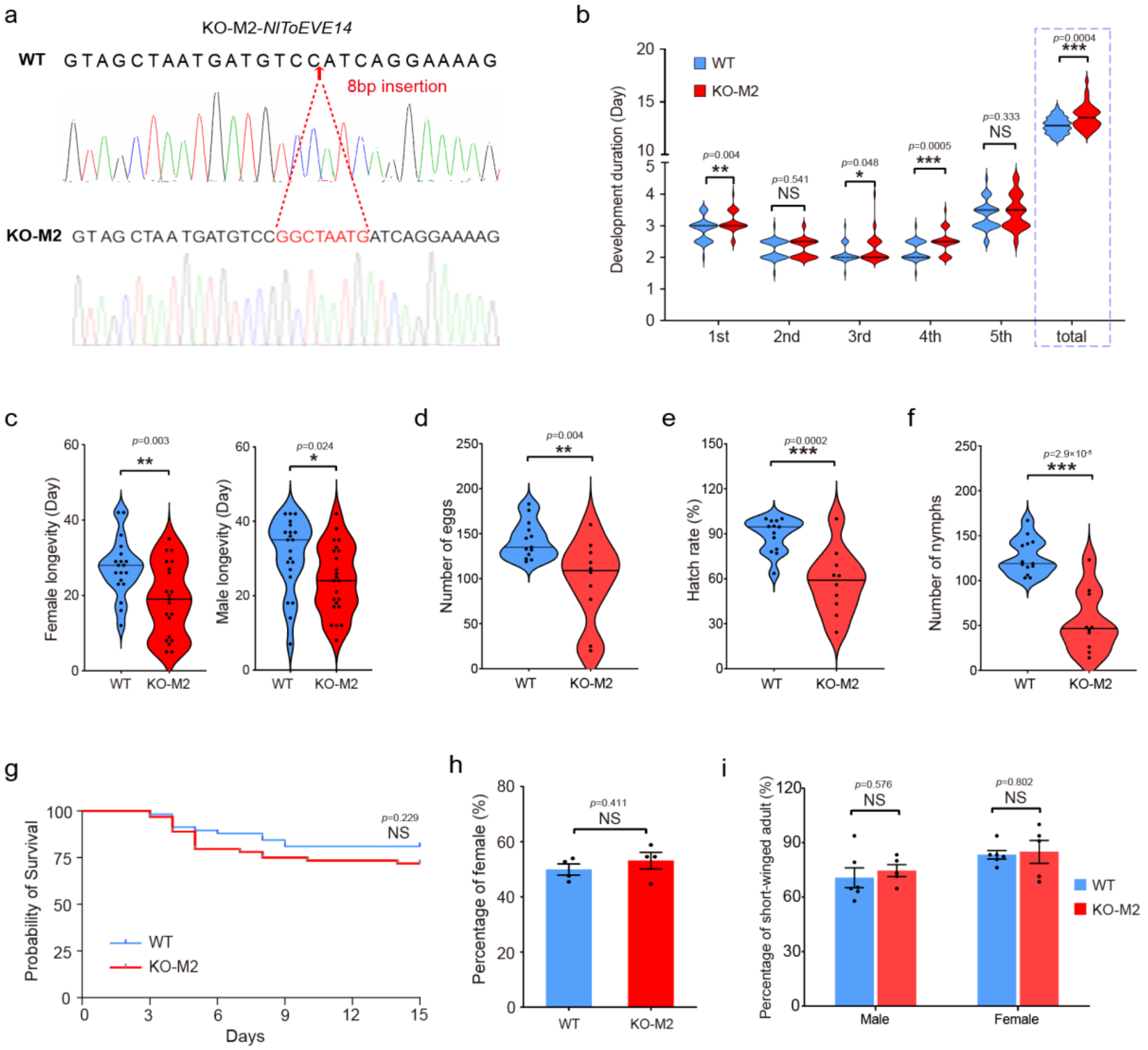

Supplemental Fig. 8: Investigation of the biological roles of NlToEVE14 in *Nilaparvata lugens* with another set of NlToEVE14-knockout strains. (a) The KO-M2 strain, which is a homozygous mutant of *N. lugens* with an 8 bp insertion in NlToEVE14. (b) The duration of the nymph development stages of *N. lugens* was significantly extended in the KO-M2 strain compared to the wild-type strain (WT). n= 42 and 36 individuals in WT and KO-M2, respectively. (c) Knockout of NlToEVE14 (KO-M2) reduced the longevity of both male and female adult *N. lugens*. n= 19, 21, 21, and 23 individuals in WT female, WT male, KO-M2 female and KO-M2 male, respectively. In the KO-M2 strain, the fecundity of *N. lugens* was significantly decreased compared to that of WT, including the number of eggs (d), the hatch rate (e), and the number of nymph offsprings (f). n= 13 and 10 independent biological replicates in WT and KO-M2, respectively. Effects of NlToEVE14 knockout on the survival rate of nymphs (g), the percentage of females (h), and the percentage of short-winged morphs (i) in the KO-M2 and WT strains. For survival analysis in (a), n= 58 and 64 individuals in WT and KO-M2, respectively; for sex analysis, n= 4 independent biological replicates; for wing analysis, n= 6

independent biological replicates. Bars in violin plots correspond to the medians. Data in (h) and (i) are presented as mean values ±SEM. *P*-values were determined by two-tailed unpaired Student's *t* test. $*P < 0.05$; $**P < 0.01$; $***P < 0.001$; NS, not significant. Source data are provided as a Source Data file.

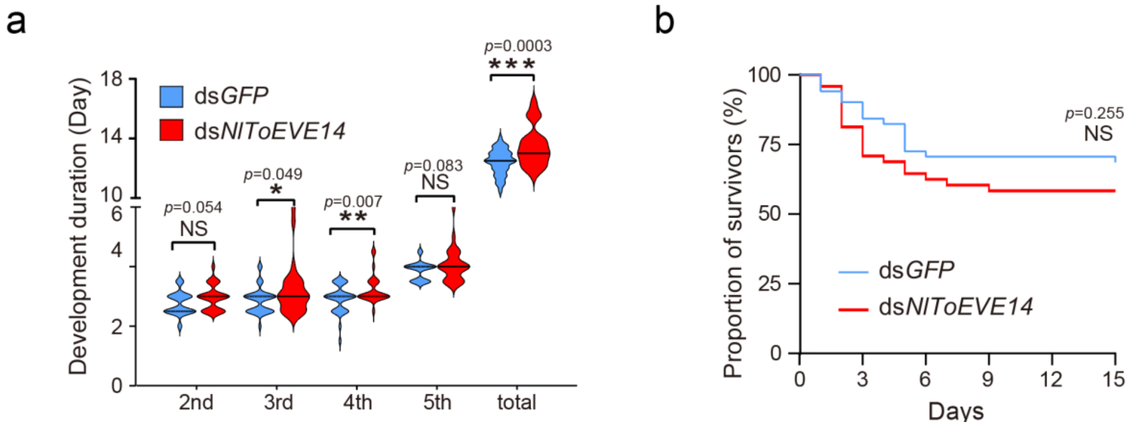

Supplemental Fig. 9: Effects of NlToEVE14 knockdown on the development duration (a) and survival rate (b) of *Nilaparvata lugens* when ds*NlToEVE14* was injected into individual planthoppers compared to the control (ds*GFP*). n= 36 and 27 individuals in ds*GFP* and ds*NlToEVE14* treatments, respectively. Bars in violin plots correspond to the medians. *P*-values in (a) were determined by two-tailed unpaired Student's *t* test. while *P*-values in (b) was determined by log-rank test. $*P < 0.05$; $**P < 0.01$; $***P < 0.001$; NS, not significant. Source data are provided as a Source Data file.

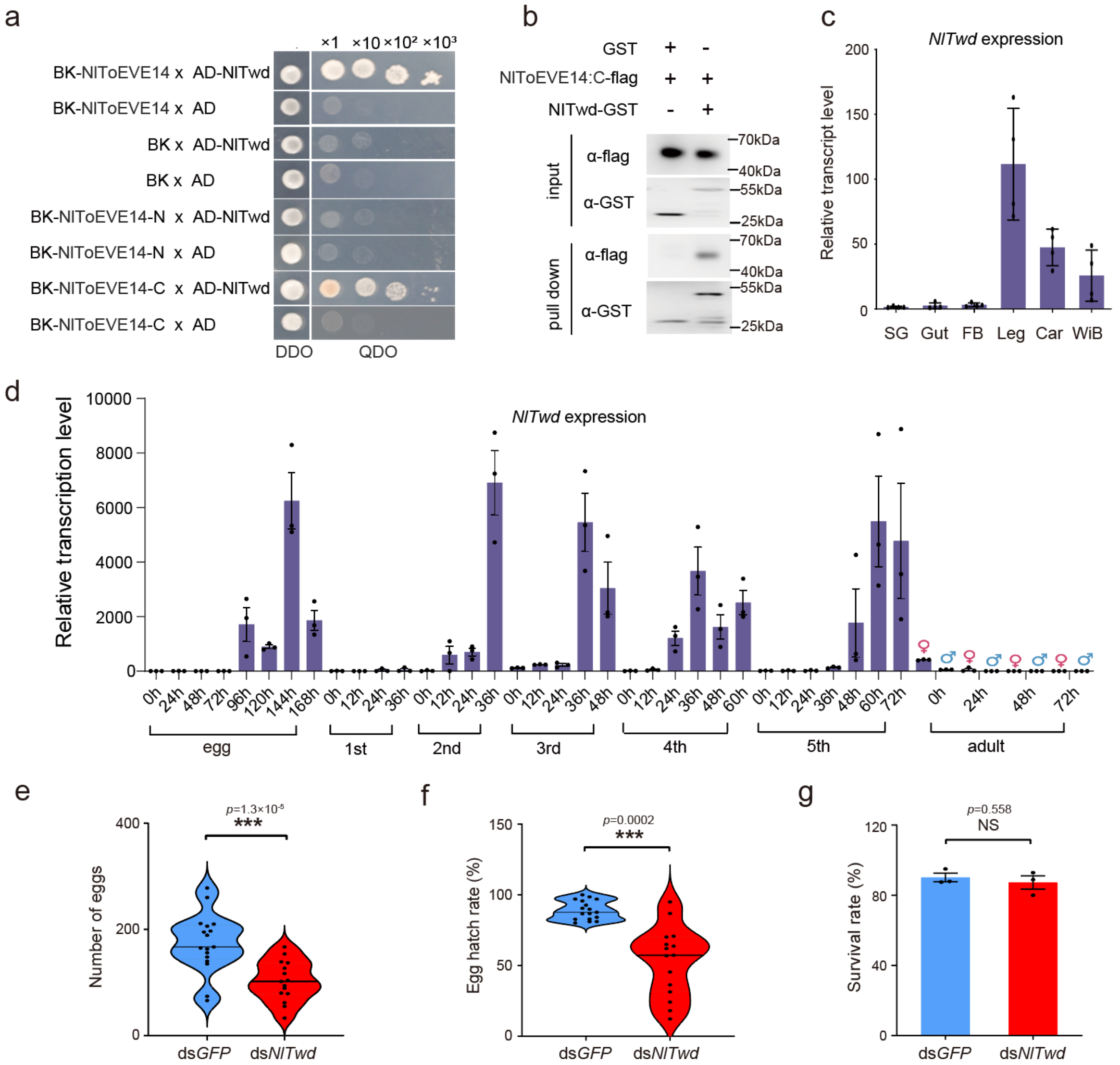

Supplemental Fig. 10: NlTwd, a NlToEVE14-associated protein, also plays essential roles in the development and fecundity of *Nilaparvata lugens*. (a) Yeast-two-hybrid assays presenting the interaction between NlToEVE14 and NlTwd. The different combinations of constructs transformed into yeast cells were grown on the selective medium SD/-Trp/-Leu (DDO), and the interactions were tested with SD/-Trp/-Leu/-His/-Ade (QDO). (b) A GST pull-down assay confirmed the interaction between NlToEVE14 and *NlTwd*. Transcript expression profiles of NlTwd in different tissues (c) and developmental stages (d) of *N. lugens*. Abbreviations, SG: Salivary gland; FB, Fat body; Car: Carcass; WiB: Wing buds. Data in (c) and (d) are presented as mean values ±SEM. n=4 and 3 independent biological replicates in (c) and (d), respectively. Effects of NlTwd knockdown on the number of eggs (e), egg hatch rate (f), and survival rate (g) of *N. lugens*, when individual planthoppers were injected with ds*NlTwd*, compared to the control (ds*GFP*). In (e) and (f), n= 19 and 15 individuals in ds*GFP* and ds*NlTwd*, respectively; in (g), n= 3 independent biological replicates. Bars in (e) and (f)

correspond to the medians. Data in (g) are presented as mean values ±SEM. The experiments in (a) and (b) were repeated two times with the similar results. *P*-values in (e), (f) and (g) were determined by two-tailed unpaired Student's *t* test. ***$P < 0.001$; NS, not significant. Source data are provided as a Source Data file.

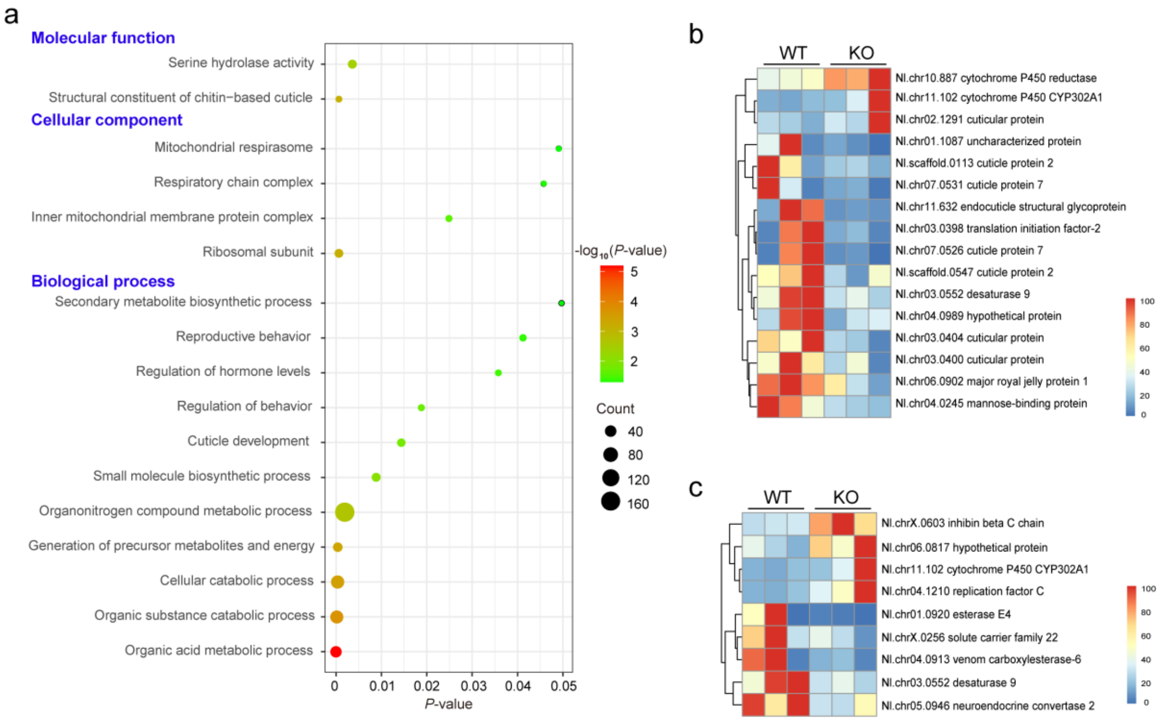

Supplemental Fig. 11: Functional analysis of differentially expressed genes (DEGs) between wild-type (WT) and NlToEVE14 knockout (KO-M1) strains. (a) Gene Ontology (GO) enrichment analysis of DEGs. The enriched GO terms were classified into three categories: cellular components, molecular functions, and biological processes. Enriched *P*-values were calculated using a one-sided hypergeometric test with TBtools software. (b, c) Heatmap analysis of DEGs associated with cuticle development (b) and regulation of hormone levels (c). The maximum transcript per million (TPM) value of each gene was set as 100. The relative transcript levels of each gene in each sample were determined accordingly. Source data are provided as a Source Data file.

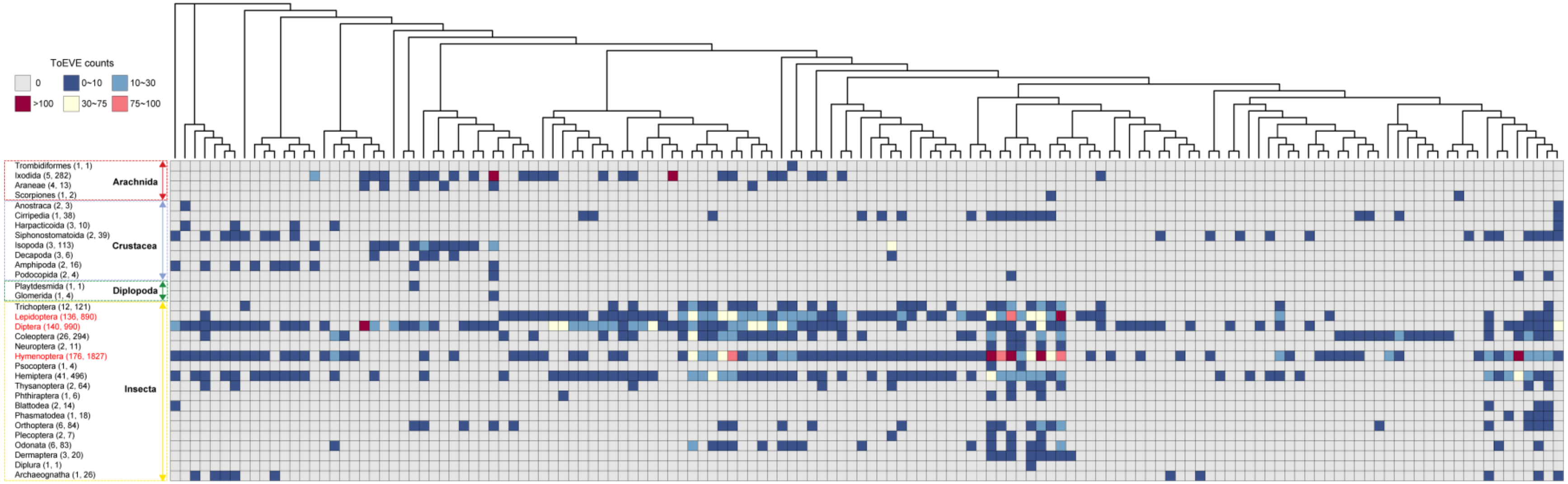

Supplemental Fig. 12: Heatmap displaying the number of ToEVEs investigated in the genomes of the arthropod taxa Arachnida, Crustacea, Diplopoda, and Insecta. Orders of arthropods are listed on the left of the main heatmap with the numbers of genomes and discovered ToEVEs in brackets. A phylogenetic tree representing the taxonomy of cognate exogenous toti/toti-like viruses is presented above the main heatmap. The three orders of Insecta (Lepidoptera, Diptera, and Hymenoptera) with the largest numbers of insect genomes are marked with red font. Source data are provided as a Source Data file.

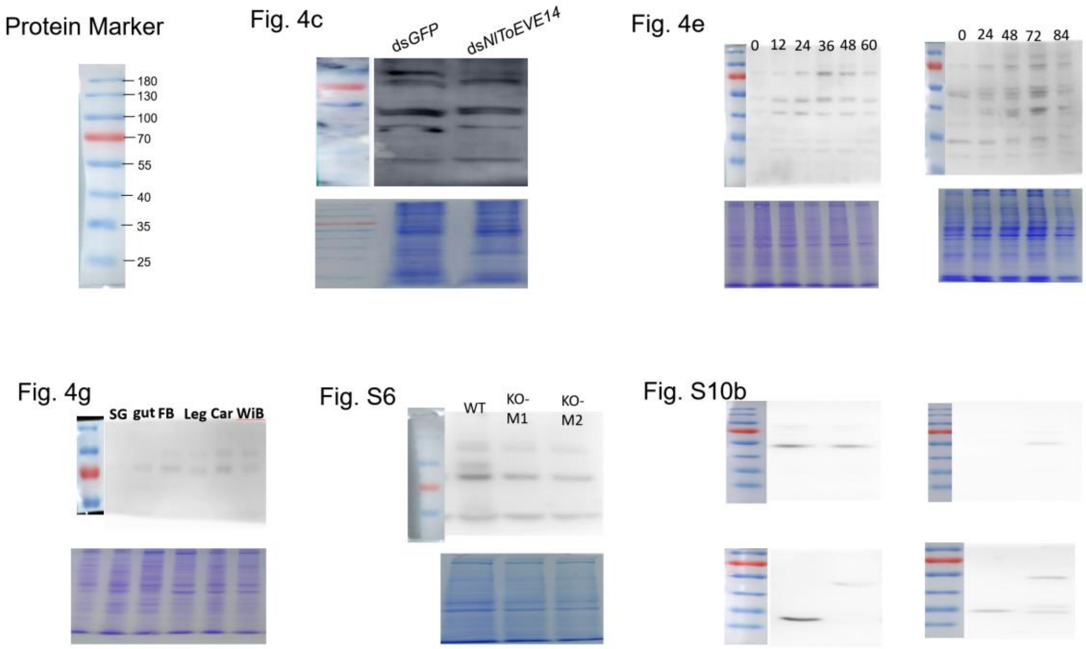

Supplemental Fig. 13: Original images for blots and gels in Figures and Supplementary Figures.

Supplemental Table 1. Identification of endogenous toti-like viral elements (ToEVEs) in the genomes of the three rice planthopper species[1,2].

ToEVE names	Length	Chr Scaffold	Chr position (Orientation)	Homologous totiviruses	Protein hits (aa position)	Identity	E-value
Sogatella furcifera ToEVE1 (SfToEVE1)	2944 nt	Chr7	32,055,107-32,054,052 (-)<br>32,055,433-32,055,958 (+)<br>32,056,331-32,056,995 (+)	NlToLV1	CP (962-1302)<br>CP (483-658)<br>CP (695-905)	35.41%<br>27.27%<br>32.43%	3.2e-64<br>3.9e-16<br>3.2e-29
SfToEVE2	1020 nt	Chr8	17,350,607-17,351,626 (+)	NlToLV1	RdRP (211-533)	48.92%	1.7e-106
SfToEVE3a*<br>SfToEVE3b*	822 nt<br>709 nt	Chr8<br>Chr11	21,854,812-21,855,633 (+)<br>16,941,783-16,942,491 (+)	NlToLV1	RdRP (651-924)	42.70%	5.5e-76
Laodelphax striatellus ToEVE1 (LsToEVE1)	4019nt	Chr1	21,210,320-21,211,415 (+)<br>21,211,776-21,213,953 (+)<br>21,213,992-21,214,338 (+)	NlToLV1	CP (246-644)<br>CP (693-1454)<br>RdRP (135-250)	27.95%<br>37.86%<br>42.24%	2.1e-29<br>1.1e-152<br>2.3e-26
LsToEVE2	806 nt	Chr2	26,126,628-26,126,856 (+)<br>26,126,847-26,127,433 (+)	NlToLV1	RdRP (427-502)<br>RdRP (499-694)	50.00%<br>62.76%	1.9e-87<br>1.9e-87
LsToEVE3	1468 nt	ChrX	13,024,430-13,024,286 (-)<br>13,024,942-13,024,460 (-)<br>13,025,331-13,025,119 (-)<br>13,025,549-13,025,292 (-)<br>13,025,753-13,025,617 (-)	NlToLV1	CP (679-725)<br>CP (736-895)<br>CP (957-1029)<br>CP (1015-1095)<br>CP (1119-1164)	36.74%<br>39.16%<br>45.21%<br>37.08%<br>41.30%	4.7e-30<br>4.7e-30<br>1.1e-12<br>1.6e-16<br>1.6e-16
LsToEVE4	446 nt	ChrX	13,524,451-13,524,157 (-)<br>13,524,602-13,524,496 (-)	NlToLV1	RdRP (263-366)<br>RdRP (386-421)	36.54%<br>41.67%	5.1e-15<br>5.1e-15
LsToEVE5	636 nt	ChrX	19,025,600-19,024,965 (-)	NlToLV1	RdRP (427-638)	55.66%	4.9e-83
LsToEVE6	1140 nt	ChrX	23,167,617-23,167,521 (-)<br>23,168,659-23,167,614 (-)	NlToLV1	CP (776-807)<br>CP (807-1151)	59.38%<br>46.18%	3.1e-10<br>8.4e-97
LsToEVE7	1728 nt	WOVE01008883.1	8495-10,222 (+)	NlToLV1	CP (332-941)	29.57%	5.7e-69
LsToEVE8	3527 nt	WOVE01012678.1	429-971 (+)<br>2039-3660 (+)	NlToLV1	CP (483-664)<br>CP (672-1202)	23.63%<br>36.25%	8.0e-12<br>2.7e-98

LsToEVE9	672 nt	WOVE01029400.1	1-672 (+)	NlToLV1	CP (916-1123)	35.72%	2.2e-37
Nilaparvata lugens ToEVE1 (NlToEVE1)	4100 nt	Chr1	28,369,231-28,368,434 (-)	NlToLV1	RdRP (659-911)	34.64%	3.7e-39
			28,369,830-28,368,997 (-)		RdRP (437-720)	50.70%	4.6e-140
			28,370,728-28,369,820 (-)		RdRP (137-440)	43.79%	4.6e-140
			28,371,340-28,370,756 (-)		CP (1248-1446)	28.00%	6.4e-43
			28,372,064-28,371,426 (-)		CP (1025-1220)	33.80%	6.4e-43
			28,372,534-28,372,031 (-)		CP (857-1025)	36.47%	1.2e-23
NlToEVE2	1287 nt	Chr2	96,095,745-96,094,459 (-)	NlToLV1	RdRP (431-859)	83.45%	0
NlToEVE3	1050 nt	Chr2	98,167,308-98,168,357 (+)	NlToLV1	CP (1057-1404)	31.73%	1.2e-52
NlToEVE4	605 nt	Chr2	98,175,873-98,176,477 (+)	NlToLV1	RdRP (295-467)	45.66%	1.9e-47
NlToEVE5	755 nt	Chr2	98,181,087-98,181,332 (+)	NlToLV1	RdRP (127-210)	40.00%	1.9e-15
			98,181,308-98,181,841 (+)		RdRP (199-377)	41.44%	2.6e-42
NlToEVE6	3969 nt	Chr3	26,570,508-26,571,362 (+)	NlToLV1	CP (672-957)	31.60%	1.1e-33
			26,571,319-26,572,083 (+)		CP (941-1189)	34.62%	3.4e-42
			26,572,092-26,572,361 (+)		CP (1193-1282)	29.03%	3.4e-42
			26,572,662-26,573,120 (+)		CP (1297-1459)	25.15%	7.2e-10
			26,572,943-26,574,040 (+)		RdRP (80-485)	36.32%	1.6e-100
			26,574,072-26,574,476 (+)		RdRP (497-631)	48.15%	1.6e-100
NlToEVE7	10,814 nt	Chr3	67,078,503-67,078,426 (-)	NlToLV1	RdRP (515-540)	65.38%	3.9e-90
			67,079,660-67,078,515 (-)		RdRP (96-510)	40.38%	3.9e-90
			67,080,445-67,079,561 (-)		CP (1154-1436)	30.90%	8.3e-88
			67,081,220-67,080,453 (-)		CP (895-1146)	36.88%	8.3e-88
			67,081,666-67,081,219 (-)		CP (745-891)	37.33%	8.3e-88
			67,088,457-67,088,115 (-)		CP (672-784)	33.91%	8.1e-11
			67,089,239-67,088,508 (-)		CP (312-600)	26.01%	3.6e-21
NlToEVE8	5166 nt	Chr5	59,246,558-59,245,440 (-)	NlToLV1	RdRP (480-843)	45.58%	2.8e-141
			59,246,772-59,246,563 (-)		RdRP (405-474)	57.14%	2.8e-141
			59,246,894-59,246,763 (-)		RdRP (360-403)	38.64%	2.8e-141
			59,247,712-59,246,951 (-)		RdRP (92-339)	30.59%	2.8e-141
			59,248,277-59,247,903 (-)		CP (1215-1339)	24.00%	4.3e-27

			59,248,632-59,248,264 (-)<br>59,249,399-59,248,623 (-)<br>59,250,605-59,249,748 (-)		CP (1099-1216)<br>CP (698-945)<br>CP (312-659)	37.10%<br>29.39%<br>25.78%	4.3e-27<br>1.7e-21<br>1.0e-21
NlToEVE9	916 nt	Chr6	48,494,324-48,494,136 (-)<br>48,494,458-48,494,315 (-)<br>48,495,051-48,494,479 (-)	NlToLV1	CP (1134-1194)<br>CP (1089-1136)<br>CP (889-1074)	33.33%<br>35.42%<br>37.24%	1.1e-39<br>1.1e-39<br>1.1e-39
NlToEVE10	625 nt	Chr7	16,056,190-16,055,909 (-)<br>16,056,533-16,056,189 (-)	NlToLV1	CP (337-427)<br>CP (219-331)	50.55%<br>61.95%	1.4e-52<br>1.4e-52
NlToEVE11	2767 nt	Chr7	51,755,628-51,752,846 (-)	NlToLV1	RdRP (1-922)	79.50%	0
NlToEVE12	504 nt	Chr7	59,852,795-59,852,292 (-)	NlToLV1	RdRP (688-855)	44.05%	2.8e-49
NlToEVE13	2059 nt	Chr7	43,665,305-43,665,790 (+)<br>43,665,784-43,667,052 (+)<br>43,667,034-43,667,363 (+)	SfToLV2	CP (209-371)<br>CP (368-795)<br>CP (791-891)	27.27%<br>28.86%<br>29.06%	1.9e-57<br>1.9e-57<br>1.9e-57
NlToEVE14	2034 nt	Chr7	55,455,324-55,457,357 (+)	SfToLV2	CP (203-891)	29.00%	2.7e-79
NlToEVE15	2289 nt	ChrX	6,417,014-6,414,726 (-)	NlToLV1	RdRP (163-925)	82.70%	0
NlToEVE16	645 nt	ChrX	6,906,320-6,905,676 (-)	NlToLV1	RdRP (401-617)	48.65%	1.1e-48
NlToEVE17	621 nt	ChrX	14,408,818-14,409,438 (+)	NlToLV1	CP (219-423)	60.87%	4.4e-73
NlToEVE18	4875 nt	ChrX	9,940,515-9,940,805 (+)<br>9,940,780-9,941,454 (+)<br>9,941,445-9,942,950 (+)<br>9,942,611-9,944,761 (+)<br>9,944,742-9,945,389 (+)	NlToLV1	CP (627-723)<br>CP (716-942)<br>CP (940-1441)<br>RdRP (1-717)<br>RdRP (710-925)	63.92%<br>68.28%<br>70.12%<br>69.32%<br>80.09%	5.3e-34<br>0<br>0<br>0<br>1.6e-114
NlToEVE19	762 nt	WOVF01001719.1	15,358-16,119 (+)	NlToLV1	RdRP (137-388)	39.76%	7.6e-65
NlToEVE20	763 nt	WOVF01002097.1	2-346 (+)<br>340-684 (+)<br>687-764 (+)	NlToLV1	RdRP (395-509)<br>RdRP (508-622)<br>RdRP (624-649)	73.91%<br>86.96%<br>92.31%	5.7e-122<br>5.7e-122<br>5.7e-122

NlToEVE21	1440 nt	WOVF01006636.1	3-479 (+)<br>482-787 (+)<br>754-1308 (+)<br>1308-1442 (+)	NlToLV1	RdRP (139-299)<br>RdRP (301-402)<br>RdRP (392-577)<br>RdRP (578-622)	42.68%<br>69.61%<br>65.44%<br>82.22%	2.7e-73<br>2.7e-73<br>5.1e-87<br>5.1e-87
NlToEVE22	2232 nt	WOVF01006191.1	4156-1925 (-)	NlToLV1	CP (12-755)	61.29%	0

[1] Abbreviation: Chr, Chromosome; NlToLV1, Nilaparvata lugens toti-like virus 1 (Accession: ON402804); SfToLV2, Sogatella furcifera toti-like virus 2 (Accession: ON402806); CP: Coat protein; RdRP, RNA-dependent RNA polymerase; +: The orientation of ToEVE is in the same direction with the predicted amino acid of the viral protein; -: The orientation of ToEVE is reverse complemented with the predicted amino acid of the viral protein; * indicated that there are more than one Chromosome integration for the same ToEVE.

[2] The sequences of ToEVEs are provided in a Source Data file.

Supplemental Table 2: Orthology analysis of endogenous toti-like viral elements (ToEVEs) in three planthopper species

Group	ToEVEs
Group-1	LsToEVE1, NlToEVE7, NlToEVE8, NlToEVE1, NlToEVE6, NlToEVE18, SfToEVE1
Group-2	NlToEVE11, NlToEVE15
Group-3	NlToEVE13, NlToEVE14

Supplemental Table 3. Transcriptome information of three planthopper species retrieved from the NCBI SRA.

Species	Accession number	Size (Gb)	Institute[a]	Stage[b]	Tissue[b]	Description
Nilaparvata lugens	DRR223269	4.4	NARO	NP	NP	VN09-3 Vietnam R strain selected by imidacloprid
	DRR223268	4.0	NARO	NP	NP	VN09-4 Vietnam R strain selected by imidacloprid
	DRR223267	3.6	NARO	NP	NP	VN09-5 Vietnam R strain selected by imidacloprid
	DRR223266	3.7	NARO	NP	NP	VN09-1 R strain
	DRR223265	3.7	NARO	NP	NP	Vietnam VN09-1 R strain
	DRR223264	3.8	NARO	NP	NP	Vietnam VN09-1 R strain
	DRR223259	3.7	NARO	NP	NP	R strain collected in Kumamoto prefecture in 2014
	DRR223258	3.3	NARO	NP	NP	R strain collected in Kumamoto prefecture in 2014
	DRR223260	4.5	NARO	NP	NP	R strain collected in Kumamoto prefecture in 2014
	DRR223254	3.5	NARO	NP	NP	Izumo S strain
	DRR223253	3.6	NARO	NP	NP	Izumo S strain
	DRR223252	3.6	NARO	NP	NP	Izumo S strain
	DRR016171	6.0	NIAS	Nymph, adult	NP	I87i strain
	DRR016172	3.6	NIAS	Nymph, adult	NP	C89i strain
	SRR10090677	7.0	ZJU	5th instar	Gut	Transferred from rice to wheat
	SRR10090685	6.7	ZJU	5th instar	Gut	Colonized on rice
	SRR10090686	6.8	ZJU	5th instar	Gut	Colonized on rice
	SRR10090687	7.2	ZJU	5th instar	Gut	Colonized on rice
	SRR10090688	7.0	ZJU	5th instar	Gut	Transferred from rice to wheat
	SRR10090689	6.7	ZJU	5th instar	Gut	Transferred from rice to wheat
	SRR10353316	6.9	SYSU	Embryo	Whole body	Embryos in 150h AEL
	SRR10353317	7.3	SYSU	Embryo	Whole body	Embryos in 150h AEL

	SRR10353318	7.5	SYSU	Embryo	Whole body	Embryos in 96h AEL
	SRR12460891	7.2	NJAU	NP	Whole body	Treated with dsGFP
	SRR12460892	7.3	NJAU	NP	Whole body	Treated with dsGFP
	SRR12460896	6.7	NJAU	NP	Whole body	Treated with dsGFP
	SRR8933211	3.7	CNRRI	NP	Whole body	Feed on TN1
	SRR8933212	4.6	CNRRI	NP	Whole body	Feed on TN1
	SRR8933214	4.9	CNRRI	NP	Whole body	Feed on Mudgo
	SRR8437756	5.9	WHU	Female adult	Head, forelegs	Feed on susceptible Nipponbare plant
	SRR8437757	6.1	WHU	Female adult	Head, forelegs	Feed on susceptible Nipponbare plant
	SRR8437758	6.7	WHU	Female adult	Head, forelegs	Feed on susceptible Nipponbare plant
	SRR8437760	6.0	WHU	Female adult	Head, forelegs	Feed on susceptible Nipponbare plant
	SRR8437761	5.6	WHU	Female adult	Head, forelegs	Feed on susceptible Nipponbare plant
	SRR8437762	6.9	WHU	Female adult	Head, forelegs	Feed on susceptible Nipponbare plant
	SRR7299433	5.7	JAAS	3rd instar	Whole body	Treated with CPF
	SRR7299371	5.5	JAAS	3rd instar	Whole body	Treated with CPF
	SRR7299348	6.2	JAAS	3rd instar	Whole body	Treated with CPF
	SRR7299271	5.4	JAAS	3rd instar	Whole body	Treated with IMI
	SRR7293611	6.1	JAAS	3rd instar	Whole body	Treated with IMI
	SRR7339932	7.5	JAAS	3rd instar	Whole body	Treated with IMI
	SRR7339787	6.8	JAAS	3rd instar	Whole body	Treated with CPF and IMI
	SRR7311307	7.2	JAAS	3rd instar	Whole body	Treated with CPF and IMI
Laodelphax striatellus	SRR12076778	12.8	JAAS	NP	Midgut	Feed on uninfected rice
	SRR12076777	15.8	JAAS	NP	Midgut	Feed on uninfected rice
	SRR12076776	14	JAAS	NP	Midgut	Feed on uninfected rice
	SRR12076775	13.4	JAAS	NP	Midgut	Feed on RBSDV infected rice

SRR12076774	15	JAAS	NP	Midgut	Feed on RBSDV infected rice
SRR12076773	14.8	JAAS	NP	Midgut	Feed on RBSDV infected rice
SRR10090678	7.5	ZJU	5th instar	Gut	Colonized on wheat
SRR10090679	7.5	ZJU	5th instar	Gut	Colonized on wheat
SRR10090680	7.9	ZJU	5th instar	Gut	Colonized on wheat
SRR10090681	7.2	ZJU	5th instar	Gut	Colonized on rice
SRR10090682	8.4	ZJU	5th instar	Gut	Colonized on rice
SRR10090684	8.3	ZJU	5th instar	Gut	Colonized on rice
SRR10090690	7.6	ZJU	5th instar	Gut	Transferred from rice to wheat
SRR10090691	7.5	ZJU	5th instar	Gut	Transferred from rice to wheat
SRR7091177	9.1	CAAS	Female adult	Ovary	RSV-free
SRR7091178	11.2	CAAS	Female adult	Ovary	Infected with RSV
SRR7091179	9.9	CAAS	Female adult	Ovary	RSV-free
SRR7091180	7.8	CAAS	Female adult	Ovary	Infected with RSV
SRR5814450	15.1	CAU	3rd instar	Hindgut	Infected with BYSMV
SRR5814451	8.6	CAU	3rd instar	Hindgut	Infected with BYSMV
SRR5814452	10.7	CAU	3rd instar	Hindgut	BYSMV-free
SRR5814453	12.7	CAU	3rd instar	Hindgut	BYSMV-free
SRR4075605	18.1	NJAU	Adult	Testes	Wolbachia-free
SRR4075602	15.5	NJAU	Adult	Testes	Wolbachia-free
SRR4075600	12.6	NJAU	Adult	Testes	Infected with Wolbachia
SRR4075914	29.7	NJAU	Adult	Testes	Infected with Wolbachia
SRR1617628	4.6	CAS	Adult	Salivary gland	RSV-free
SRR1619428	4.7	CAS	Adult	Salivary gland	Infected with RSV
SRR1617623	4.6	CAS	Adult	Gut	RSV-free

	SRR1617620	4.1	CAS	Adult	Gut	Infected with RSV
	SRR1614218	4.6	CAS	Adult	Salivary gland	RSV-free
	SRR1614222	4	CAS	Adult	Salivary gland	Infected with RSV
	SRR1617622	4.2	CAS	Adult	Gut	RSV-free
	SRR1617617	5.5	CAS	Adult	Gut	Infected with RSV
	SRR5816380	7.3	CAS	NP	Whole body	NP
	SRR5816381	7	CAS	Nymph	NP	NP
	SRR5816383	7.2	CAS	Egg	Whole body	NP
Sogatella furcifera	SRR10230344	7.1	ZJU	5th instar	Gut	Colonized on rice
	SRR10230345	7.1	ZJU	5th instar	Gut	Colonized on rice
	SRR10230346	6.8	ZJU	5th instar	Gut	Transferred from rice to wheat
	SRR10230347	7.1	ZJU	5th instar	Gut	Transferred from rice to wheat
	SRR10230348	6.6	ZJU	5th instar	Gut	Transferred from rice to wheat
	SRR10230349	7.1	ZJU	5th instar	Gut	Colonized on wheat
	SRR10230350	6.8	ZJU	5th instar	Gut	Colonized on wheat
	SRR10230351	6.8	ZJU	5th instar	Gut	Colonized on wheat
	SRR10230352	6.9	ZJU	5th instar	Gut	Colonized on rice
	SRR363380	2.4	ZJU	NP	NP	SRBSDV-free
	SRR3990920	5.1	USTC	NP	Whole body	NP
	SRR3990986	9.7	USTC	NP	Whole body	NP
	SRR3990981	10	USTC	NP	Whole body	NP
	SRR3990923	11.3	USTC	NP	Whole body	NP
	SRR3990922	8.9	USTC	NP	Whole body	NP
	SRR3990921	10.3	USTC	NP	Whole body	NP
	SRR7772175	3.1	USTC	Adult	Whole body	Infected with SRBSDV

SRR7772160	9.1	USTC	Adult	Whole body	Infected with SRBSDV
SRR7772177	3.9	USTC	Adult	Whole body	SRBSDV-free
SRR7772176	4.8	USTC	Adult	Whole body	SRBSDV-free
SRR5980155	6	GZU	5th instar	Whole body	Treated with Deltamethrin
SRR5980156	6	GZU	5th instar	Whole body	Treated with Triazophos
SRR5980157	5.9	GZU	5th instar	Whole body	Treated with Imidacloprid
SRR5980158	6	GZU	5th instar	Whole body	No pesticide treatment
SRR5980154	6.7	GZU	5th instar	Whole body	No pesticide treatment
SRR11729958	12.1	CNRRI	NP	Whole body	Collected in Fushun, China
SRR11658058	13.8	CNRRI	NP	Whole body	Collected in Fuyang, China
SRR9986018	8.9	SCAU	3rd instar	NP	Collected in Sichuan, China
SRR9986019	8.5	SCAU	3rd instar	NP	Collected in Sichuan, China
SRR9986020	8.4	SCAU	3rd instar	NP	Collected in Sichuan, China
SRR9986021	7.6	SCAU	3rd instar	NP	Collected in Sichuan, China
SRR9986022	8.1	SCAU	3rd instar	NP	Collected in Sichuan, China
SRR9986023	8.9	SCAU	3rd instar	NP	Collected in Sichuan, China
SRR8904472	6.4	SYSU	5th instar	Whole body	Female with long winged bud
SRR8904473	6.7	SYSU	5th instar	Whole body	Male with long winged bud
SRR8904474	6.6	SYSU	5th instar	Whole body	Male with long winged bud
SRR8904475	5.9	SYSU	5th instar	Whole body	Male with long winged bud
SRR8904476	6.4	SYSU	5th instar	Whole body	Female with long winged bud
SRR8904477	6.7	SYSU	5th instar	Whole body	Female with long winged bud
SRR3211109	15	CAAS	Adult	Salivary gland	NP

[a] NARO, Institute of Agrobiological Sciences, National Agriculture and Food Research Organization; NIAS, National Institute of Agrobiological Sciences; ZJU, Zhejiang University; SYSU, Sun Yat-sen University; NJAU, Nanjing Agricultural University; CNRRI, China National Rice Research Institute; WHU,

Wuhan University; JAAS, Jiangsu Academy of Agricultural Sciences; CAAS, Chinese Academy of Agricultural Sciences; CAU, China Agricultural University; CAS, Chinese Academy of Sciences; USTC, University of Science and Technology of China; GZU, Guizhou University; SCAU, Sichuan Agricultural University.

[b] NP, not provided

Supplemental Table 4. Identification of endogenous toti-like viral elements (ToEVEs) from selected representative arthropod genomes.

Species	Common Name	Order/Class	Genome size	NCBI Accession	Assembly level	Number of ToEVEs [1]
Agrilus planipennis	Emerald ash borer	Coleoptera	353M	GCF_000699045.2	Scaffold	6
Sitophilus oryzae	Rice weevil		770M	GCF_002938485.1	Scaffold	2
Aedes aegypti	Yellow fever mosquito	Diptera	1279M	GCF_002204515.2	Chromosome	8
Aedes albopictus	Asian tiger mosquito		2538M	GCF_006496715.1	Scaffold	23
Drosophila navojoa	Fruit fly		147M	GCF_001654015.2	Scaffold	1
Drosophila yakuba	Fruit fly		166M	GCF_000005975.2	Chromosome	1
Acyrthosiphon pisum	Pea aphid	Hemiptera	541M	GCF_005508785.1	Chromosome	4
Bemisia tabaci (MEAM1)	Silverleaf whitefly		615M	GCA_001854935.1	Scaffold	11
Diaphorina citri	Asian citrus psyllid		486M	GCA_000475195.1	Scaffold	1
Laodelphax striatellus	Small brown planthopper		540M	GCA_014465815.1	Chromosome	9
Nilaparvata lugens	Brown planthopper		1088M	GCA_014356525.1	Chromosome	22
Sogatella furcifera	White-backed planthopper		656M	GCA_014356515.1	Chromosome	3
Trialeurodes vaporariorum	Greenhouse whitefly		787M	GCA_011764245.1	Scaffold	2
Belonocnema treatae	Oak gall wasps	Hymenoptera	1539M	GCF_010883055.1	Chromosome	4
Bombus terrestris	Bumblebee		249M	GCF_000214255.1	Chromosome	1
Ceratina calcarata	Carpenter bee		200M	GCF_001652005.1	Scaffold	11
Formica exsecta	Narrow-headed ant		278M	GCF_003651465.1	Scaffold	1
Megachile rotundata	Alfalfa leafcutting bee		273M	GCF_000220905.1	Scaffold	9
Nomia melanderi	Alkali bee		326M	GCF_003710045.1	Scaffold	8
Osmia bicornis	Red mason bee		211M	GCF_907164935.1	Scaffold	9

Pseudomyrmex gracilis	Slender twig ant		283M	GCF_002006095.1	Scaffold	9
Trichogramma pretiosum	Parasitoid wasp		188M	GCF_000599845.2	Scaffold	1
Wasmannia auropunctata	Little fire ant		324M	GCF_000956235.1	Scaffold	1
Bombyx mori	Domestic silk moth	Lepidoptera	460M	GCF_014905235.1	Chromosome	6
Bombyx mandarina	Wild silk moth		398M	GCF_003987935.1	Scaffold	9
Plutella xylostella	Diamondback moth		343M	GCA_019096205.1	Scaffold	1
Spodoptera litura	Tobacco cutworm		439M	GCF_002706865.1	Chromosome	1
Calopteryx splendens	Banded demoiselle	Odonata	1628M	GCA_002093875.1	Scaffold	5
Pantala flavescens	Wandering Glider		663M	GCA_020796165.1	Chromosome	1
Ischnura elegans	Blue-tailed damselfly		1723M	GCA_921293095.1	Chromosome	4
Frankliniella occidentalis	Western flower thrips	Thysanoptera	275M	GCF_000697945.2	Scaffold	8
Thrips palmi	Melon thrips		238M	GCF_012932325.1	Scaffold	2
Dermacentor silvarum	Hard tick	Arachnida	2474M	GCF_013339745.1	Chromosome	30
Rhipicephalus sanguineus	Brown dog tick		2366M	GCF_013339695.1	Chromosome	8
Rhipicephalus microplus	Cattle tick		2530M	GCF_013339725.1	Chromosome	24
Ixodes scapularis	Deer tick		2227M	GCF_016920785.2	Scaffold	3
Portunus trituberculatus	Swimming crab	Malacostraca	1005M	GCF_017591435.1	Chromosome	1

[1] The sequences of ToEVEs are provided in a Source Data file.

Supplemental Table 5. Detailed transcriptome information used to identify ToEVE transcripts of the selected representative arthropods.

Species	Accession Number	Order (Class)	Total Base (Gb)	ToEVE transcripts [1] (>200 bp)	Submitter	Description
Agrilus planipennis	SRR1791267	Coleoptera	7.6	0	Baylor College of Medicine	*Agrilus planipennis* whole organism sample APLA.00-female
	SRR8304515		3.7	0	The Ohio State University	Midgut of larvae feeding on green ash phloem
	SRR1615252		7.9	0	Natural Resources Canada	Midgut of Larve
Sitophilus oryzae	SRR4288810		8.1	1	Hefei University of Technology	N/A
	SRR15116543		6.0	0	Biologie Fonctionnelle, Insectes et Interactions	RNAseq of ovaries of day 10 *Sitophilus oryzae* females
	SRR13089456		10.5	0	Lanzhou University	Phylogenetic relationships of Cucujiformia based on transcriptomic data Raw sequence reads
Aedes aegypti	SRR11715731	Diptera	11.6	0	Queensland University of Technology	Adult uninfected female at 32℃, 7days post Chikungunya virus infection
	SRR19214202		9.2	3	Johns Hopkins School of Public Health	RNA-seq of Aedes R2d2-3 midgut
	SRR19090271		12.1	3	University of Antioquia	Adult mosquitoes
	SRR17556106		4.2	0	Laboratory of Neurogenetics and Behavior, The Rockefeller University	*Ae. aegypti* ovary 13 days post-blood-meal with eggs laid >1 week prior

Aedes albopictus	SRR13106788		22.1	0	University of Illinois at Urbana-Champaign	Effects of phytochemicals in the diet of *Aedes albopictus*
	SRR13758741		6.9	0	AMMS	RNA-Seq of Aedes albopictus C6/36 Cells
	SRR13521497		6.3	0	Armbruster Lab, Georgetown University	Linkage map anchored genome reassembly and diapause associated SNPs in Aedes albopictus
Drosophila navojoa	SRR7958874		15.9	0	Center for Research and Advanced Studies of the National Polytechnic Institute	N/A
	SRR7973855		1.9	0	Center of Research and Advanced Studies	Whole adult female
Drosophila yakuba	SRR7284675		8.0	0	University of Iowa	Female adult
	SRR10253135		8.0	0	University of California Davis	Tissue Specific Expression in Drosophila
	SRR9678428		6.5	2	The Rockefeller University	RNA-sequencing of *Drosophila yakuba* adult female brain
Acyrthosiphon pisum	SRR14670939	Hemiptera	13.6	0	University of Rochester	RNA-Seq of *acyrthosiphon pisum*: first instar wingless female wholebody
	SRR18904304		5.8	0	South China Normal University	RNA-seq of flight muscle
	SRR15212270		4.1	0	National Research Institute for Agriculture, Food and Environment (INRAE)	RNA-Seq of pea aphid heads, legs and gonads from males, sexual females and parthenogenetic adults
Bemisia tabaci	SRR13050950		11.7	0	Ningbo University	RNA virome diversity of whitefly
Diaphorina citri	SRR10352965		9.8	0	Zhongkai University of Agriculture and Engineering	Beta-cypermethrin untreated *Diaphorina citri* adults

	SRR11802724		6.4	0	South China Agricultural University	RNA-Seq of *Diaphorina citri* egg
	SRR11097188		15.1	1	University of Florida	*Diaphorina citri* Kuwayama (Hemiptera: Liviidae) in insecticide thiamethoxam resistance
Trialeurodes vaporariorum	SRR9038790		6.6	0	Shenyang agricultural university	200 adults greenhouse whiteflies not carrying Arsenophonus
	SRR10192918		6.2	1	Chinese Academy of Agricultural Science Institute	*Trialeurodes vaporariorum* isolate: IVF Genome sequencing and assembly
	SRR9309903		4.1	1	University of Exeter	Whole body
Belonocnema treatae	SRR10953995	Hymenoptera	4.9	0	University of Georgia	Female adult
	SRR10961349		10.5	0	University of Georgia	RNA-Seq Belonocnema treatae Sexual Venom Gland 2
Bombus terrestris	SRR11448239		11.1	0	Universidade de Sao Paulo	Whole body nurses pool
	SRR8935808		2.7	1	University College Cork	RNA-Seq of nematode infected bumblebee queens
	SRR6148368		3.0	1	Ghent University	Head and abdomen
Ceratina calcarata	SRR1284947		11.7	0	University of New Hampshire	Differential gene expression analyses
	SRR13282007		6.6	2	University of New Hampshire	RNA-Seq of *Ceratina calcarata* brain

	SRR6819910	11.4	5	GEO	heads of newly eclosed orphaned bees
Formica exsecta	SRR2080399	9.9	0	University of Helsinki	*Formica exsecta* Transcriptome or Gene expression
	SRR945909	7.0	0	Department of Biosciences, University of Helsinki	CocoonQueen
Megachile rotundata	SRR1582755	6.5	0	North Dakota State University	RNA-seq from post-diapause quiescent prepupae
	SRR8767057	4.8	0	United States Department of Agriculture, Agricultu	Invertebrate sample from whole *Megachile rotundata* pupa
	SRR5979999	0.8	0	USDA-ARS	*Megachile rotundata* late season diapausing
Nomia melanderi	SRR13061852	7.5	0	Cornell University	RNA seq of *Nomia melanderi* female Antennae
	SRR13061851	3.3	1	Cornell University	RNA seq of *Nomia melanderi* female Antennae
Osmia bicornis	SRR2895248	6.0	0	Rothamsted Research	*Osmia bicornis* bicornis Genome sequencing and assembly
	SRR7286081	2.4	1	University of Exeter	*Osmia bicornis* bicornis Genome sequencing and assembly
	SRR6148379	3.7	1	Ghent University	RNA-Seq of *Osmia bicornis* head and abdomen
Pseudomyrmex gracilis	SRR1743363	40.8	0	University of Chicago	*Pseudomyrmex gracilis* Genome sequencing and assembly
	SRR12918485	3.9	1	Western Sydney University	With Varroa non apiary ant
Trichogramma pretiosum	SRR5059286	10.8	0	Georgia Institute of Technology	Adult female *Trichogramma pretiosum* from the Insectary line

	ERR766212		9.5	0	Vittal Mallya Scientific Research Foundation	Trancriptome sequence of *Trichogramma pretiosum* (Wild)
	SRR1826957		4.4	0	Baylor College of Medicine	whole organism sample TPRE.00-female
Bombyx mori	SRR13802866	Lepidoptera	8.9	0	NCBI (GEO)	Precocious metamorphosis of silkworm by BmNPV infection in the latter half of the fifth instar larvae
	SRR2034847		1.8	0	NCBI (GEO)	BmN4 transfected with Ago3-endo reporter
Bombyx mandarina	SRR1619469		5.8	0	Southwest University	The middle silkgland and posterior silkgland of the wild silkworm larvae
	SRR1619431		6.3	0	Southwest University	The middle silkgland and posterior silkgland of the wild silkworm larvae
	SRR6031689		7.3	3	Chongqing University	Antennal transcriptomes of *Bombyx mandarina*
Plutella xylostella	SRR5171457		17.1	2	China Agricultural University	N/A
	SRR5616379		7.4	0	University of Cambridge	*Plutella xylostella* female soma transcriptome
	SRR3623304		7.1	1	ICAR-National Bureau of Agricultural Insect Resour	Plutella Susceptible 2
Spodoptera litura	SRR13664485		9.6	0	Sichuan Agricultural University	Transcriptome Analysis of the Resistance of *Spodoptera litura* to Diflufenuron
	SRR8772860		4.5	0	Institute of Cotton Research, Chinese Academy of A	RNA-seq of *Spodoptera litura* fed on cotton diet midgut
	SRR1982877		3.7	0	NCBI (GEO)	Transcriptome Responses of the Host *Spodoptera litura* upon

						Spodoptera litura nucleopolyhedrovirus infection
Calopteryx splendens	SRR921575	Odonata	3.9	0	Centre for Molecular Biodiversity Research	The 1KITE project: evolution of insects
Pantala flavescens	SRR1184263		1.5	0	GEO	Transcriptome of *Pantala flavescens* Fabricius
Ischnura elegans	SRR2962606		8.8	0	Lund university	Thermal gene expression and range shift
	SRR12660678		4.3	0	GEO	RNA-Seq of *Ischnura elegans* larvae
	SRR1184452		1.8	0	Nankai University	The transcriptome of *Ischnura elegans* for phylogenomic study
Frankliniella occidentalis	SRR15676027	Thysanoptera	7.5	0	Nanjing Agriculture University	RNA-Seq of *Frankliniella occidentalis* adults grown under aCO2
	SRR19795994		12.5	0	University of Cincinnati	Dehydrated Female
	SRR15207604		9.6	0	NC State University	RNAseq of *F. occidentalis* early second instar: non-infected guts
Thrips palmi	SRR8113224		8.0	0	The University of Queensland	Response of *Thrips palmi* (melon thrips) to capsicum chlorosis virus (CaCV)
	SRR11177268		6.8	0	Beijing Academy of Agriculture and Forestry Sciences	N/A
	SRR921649		2.7	0	Centre for Molecular Biodiversity Research	The 1KITE project: evolution of insects
Dermacentor silvarum	SRR10123454	Arachnida	4.6	1	Hebei Normal University	adult female

	SRR18473713		12.0	12	Beijing Institute of Microbiology & Epidemiology	adult female
	SRR17422321		14.0	0	Hainan medical University	RNA pool from 15-20 ticks
Rhipicephalus sanguineus	SRR14866272		10.4	8	Universidad Pontificia Bolivariana	*Rhipicephalus sanguineus* from canines
	ERR793488		7.8	1	UNIMIDIVET	N/A
	SRR13614649		29.7	2	USDA-Agricultural Research Service	Adult females newly molted and unfed
Rhipicephalus microplus	SRR13562174		3.2	0	Universidade Federal Rural do Rio de Janeiro	Adult females salivary glands
	SRR10740626		3.3	0	EMBRAPA Informatica Agropecuaria	RNA-Seq of cattle tick salivary gland
	SRR15831584		15.1	1	NCBI (GEO)	RdRp in *Ixodes scapularis* produce Argonaute-dependent sRNAs
Ixodes scapularis	SRR14748243		2.4	0	Wuhan Institute Of Virology,Chinese Academy Of Sciences	Transcriptome of IDE8 tick cell lines
	SRR8896911		4.8	0	National Institute of Allergy and Infectious Diseases	RNA-seq of two Borrelia burgdorferi-infected adult *Ixodes scapularis* midguts fed for 5 days treated with ds selK
Portunus trituberculatus	SRR17274363	Malacostraca	7.8	0	Ningbo University	Testis sequencing
	SRR6233346		50.1	1	Ningbo University	N/A

[1] The sequences of ToEVEs are provided in a Source Data file.

Supplementary Table S6. Primers used in this study

Genes	Forward primer (5'-3')	Reverse primer (5'-3')
**Primers used in PCR/RT-PCR**		
NlToLV1-1	GTCTTCGTAGTACTTCGCTGC	ACTGTATGCCAGGAAATCACG
NlToLV1-2	CCGTAAGTTTAAGGATTATGC	TTTGCCGTACGCAGTCATATC
NlToLV1-3	TCCACCGAGAATAGAAGGCAG	GTAAGCTCTCAGTTTGCACAG
NlToLV1-4	CGATCCCCGATACTGGTTTCC	AGCCTCATCGGTTTTACCAG
NlToLV1-5	GGAAGTCGTACGAATCCAAC	TACGCGTCAATTGTCAAGTC
NlToEVE1	GAAAAAAACTCAGACTGGGAC	GTATTGCTTCTCCTGGAGTAG
NlToEVE2	ACTCAGGGTGGATGTACTTCG	GAGAGACTACGTGAGAGAATG
NlToEVE3	GACACCCCTGATATGTCTGGT	CCCCATGAGAGTTTAACGCTGG
NlToEVE4	TTGGGAAAGAGTATTGGAGTAG	GTGTTGCAAGAAGTCGTCGTC
NlToEVE5	CACCTTCCTCAAAGCTGAGGA	CTTTGTCACTAAGATGGACCC
NlToEVE6	GGGCATAATCTAAACAACCCTG	CCTCCTCCTACAAATCACTCC
NlToEVE7	GCAATGTATCCAGTAAGGCGG	ACCCACGAGCAATTGATGGAC
NlToEVE8	CTGCATCTGTGGAACAATGTAG	CAGTCACCAATCTCATAGCCC
NlToEVE9	CGAGTCCTTTATCATCACCATC	CACCCACTCGCAGGCATTCTAG
NlToEVE10	CTCAACGAGATCATTGGTTTC	TGGAGTGTAACGGTAGCTACC
NlToEVE11	GAGATAGTAAAGCTCGAGGGC	AGGTTGGATCGTTGATGCTGG
NlToEVE12	TCCCTGACTGACTCGCTTCT	GTCTACTGCCCAGTGCATTTG

NlToEVE13	ATTGAGTGTAGCTTTACCTGC	ACTCTCCTGTTCTATGGATAC
NlToEVE14	GAATTGTCTCCTGACGAACCTG	TAGCTTGCAAATCAGAGAGATG
NlToEVE15	CGCACTGTTTCAGGTAGTACA	CTGGTGTAGAGAAAGACAAGG
NlToEVE16	TCCGGTTCGTACGATTTTCGC	TGGCTATGCTGCATCTCATG
NlToEVE17	TTCTTGGAGCATAGCGGCAG	CTACTAACCCATCAGGCTCATC
NlToEVE18	AACATCATGCCGGTTAGCTGG	GGCACGAACCATTCCTACTTC
NlToEVE19	GACGAAAACTGGTCATGGAACACG	CTACCTGTTCCAGCTTTACCA
NlToEVE20	GAACAGGGCAAATCAAGGCC	TGGCACCTTTATGGGGTTCC
NlToEVE21	CCTGTTCACTGCTAATCCCAG	CGCGGAAGATGGATCGAATTG
NlToEVE22	CAATGTTGGCGGCTCTCTTAG	CGGTTATCGCGTTTGATGGTG
LsToEVE1	ACTTGGGATGTACCATGAAGAG	ACTTGGGATGTACCATGAAGAG
LsToEVE2	ACAAAATCCGACAGCGTGGTG	TGCTCCATCTCAACCCTGTTC
LsToEVE3	TGCTCTCCGGACGTAGTGAC	TGCGAGTCCCATTCGTGAGG
LsToEVE4	GGCACCACTGTGAGAGAAATG	GATTAGCAGCACCCATTGTAC
LsToEVE5	CTACCTCTATAGCCTGAAAGTC	TGTCCTCTGTCCACGCTATTT
LsToEVE6	AGGTCGTTAGGTCTGAGAATAG	TATCACTGGTGTATTGTGGGC
LsToEVE7	GTTGGAACAGGCTGATAATAG	GCTGGCTGAAGACTTCATTCC

LsToEVE8	TGCTCTCCGGACGTAGTGAC	TTGCGAGTCCCATTCGTGAG
LsToEVE9	CCTTGCTGCTACATCTTGCTC	AGGAACTCCAGCTACGTTAGC
SfToEVE1	GTGCACGATCCAGTTGATTC	TGTTGCCGACGATAAAGCTG
SfToEVE2	TCAAAGAGTGGTACAGACGG	GTGAAGCCAAACTGTAGAACC
SfToEVE3	TGGAGACGCCAACTCAGTGTC	GCACATTATGAGATCAGCGTA
**Primers used in qPCR**		
NlToEVE14	CCATTCACAGAGGCTAGATAT	GAACCAGAACTCATCATTACG
NlActin	GTGCGTGACATCAAGGAGAAGC	GGAAGGAAGGCTGGAACAGAG
NlTwdlE	CCAATCGTACAGAAGCATATC	GGTGCCTTAATGAAGATGATC
**Primers used in double stranded RNA synthesis**		
GFP	TAATACGACTCACTATAGGGAGAATGAGTAAAGGAGAAGAACTTTTC	TAATACGACTCACTATAGGGAGATTTGTATAGTTCATCCATGCCATGT
NlToEVE14	TAATACGACTCACTATAGGGGATGTCTCTGATTGGAAAGGTC	TAATACGACTCACTATAGGGTTAGCTTTCCCGAAGGTGGTG
NlTwdIE	TAATACGACTCACTATAGGGAGGCACCAATCGTACAGAAGC	TAATACGACTCACTATAGGGGTGTCAGCTGACGCTTAGTAGG
**Primers used in vector construction**		
BK-NlToEVE14	TCAGAGGAGGACCTGCATATGATGGGCAGTTTCGGCATGCCGC	CCGCTGCAGGTCGACGGATCCCTATTGTTGTGAACTTGTGCCT
BK-NlToEVE14:N	TCAGAGGAGGACCTGCATATGATGGGCAGTTTCGGCATGCCG	CCGCTGCAGGTCGACGGATCCCCAGTGAATAACCTGCAAATC
BK-NlToEVE14:C	TCAGAGGAGGACCTGCATATGGCTAAGGCTGGACATCGT	CCGCTGCAGGTCGACGGATCCACTTGTGCCTAGACACGG

	ATC	AAA
AD-NlTwdIE	GTACCAGATTACGCTCATATGAGGGCGTTCGTTGTTCTA	CAGCTCGAGCTCGATGGATCCGTGTCAGCTGACGCTTAG TAGG
293flag-NlToEVE14:C	AAGGATGACGATGACAAGCTTATGGCTAAGGCTGGACAT CGTATC	CCTCTAGAGTCGACTGGTACCACTTGTGCCTAGACACGG AAA
**Primers used in Crispa/Cas9**		
sgRNA-NlToEVE14	TAATACGACTCACTATAGGTAGCTAATGATGTCCATCGTTT TAGAGCTAGAAATAGC	AAAAAAAGCACCGACTCGGTGCCAC
