# Supplementary material for: Co-option of a non-retroviral endogenous viral element in planthoppers
Source: Nat Commun. 2023 Nov 9;14:7264. doi: 10.1038/s41467-023-43186-2 (PMC10636211; doi:10.1038/s41467-023-43186-2)
Supplement: Supplementary file 3 — Description of Additional Supplementary Files [file 41467_2023_43186_MOESM3_ESM.pdf]

Description of Additional Supplementary Files

File Name: Supplementary Data 1
Description: Arthropod genome analyzed in this study

File Name: Supplementary Data 2
Description: ToEVEs in Arthropod genomes
