# Supplementary material for: Co-option of a non-retroviral endogenous viral element in planthoppers
Source: Nat Commun. 2023 Nov 9;14:7264. doi: 10.1038/s41467-023-43186-2 (PMC10636211; doi:10.1038/s41467-023-43186-2)
Supplement: Supplementary file 6 — Reporting Summary [file 41467_2023_43186_MOESM6_ESM.pdf]

nature portfolio

Corresponding author(s): Jian-Ping Chen
Chuan-Xi Zhang
Jun-Min Li

Last updated by author(s): Oct 24, 2023

Reporting Summary

Nature Portfolio wishes to improve the reproducibility of the work that we publish. This form provides structure for consistency and transparency in reporting. For further information on Nature Portfolio policies, see our Editorial Policies and the Editorial Policy Checklist.

Statistics

For all statistical analyses, confirm that the following items are present in the figure legend, table legend, main text, or Methods section.

n/a	Confirmed	
☐	☒	The exact sample size (*n*) for each experimental group/condition, given as a discrete number and unit of measurement
☐	☒	A statement on whether measurements were taken from distinct samples or whether the same sample was measured repeatedly
☐	☒	The statistical test(s) used AND whether they are one- or two-sided<br>*Only common tests should be described solely by name; describe more complex techniques in the Methods section.*
☒	☐	A description of all covariates tested
☒	☐	A description of any assumptions or corrections, such as tests of normality and adjustment for multiple comparisons
☐	☒	A full description of the statistical parameters including central tendency (e.g. means) or other basic estimates (e.g. regression coefficient) AND variation (e.g. standard deviation) or associated estimates of uncertainty (e.g. confidence intervals)
☐	☒	For null hypothesis testing, the test statistic (e.g. *F*, *t*, *r*) with confidence intervals, effect sizes, degrees of freedom and *P* value noted<br>*Give P values as exact values whenever suitable.*
☒	☐	For Bayesian analysis, information on the choice of priors and Markov chain Monte Carlo settings
☒	☐	For hierarchical and complex designs, identification of the appropriate level for tests and full reporting of outcomes
☒	☐	Estimates of effect sizes (e.g. Cohen's *d*, Pearson's *r*), indicating how they were calculated

Our web collection on statistics for biologists contains articles on many of the points above.

Software and code

Policy information about availability of computer code

Data collection	AI 680 image analyzer for WB visualization; Roche Light Cycler® 480 Real-Time PCR System for qPCR
Data analysis	The codes and softwares used in this study were listed below. In detail, , local BLAST (https://ftp.ncbi.nlm.nih.gov/blast/executables/blast+/LATEST) was used to identify toti-like viruses and ETLVEs in transcriptomes and genomes. Trinity software v2.8.5 was used to assemble transcriptomes. Protein conserved domains were predicted by InterPro 89.0 (http://www.ebi.ac.uk/interpro/). The MAFFT v7.505 was used to perform sequence alignment, and the poorly aligned sequences were removed using Gblocks version 0.91b. The OrthoMCL v2.0.9 was used to identify orthologous groups of three planthoppers. Markov chain clustering MCL14–137 was used to identify protein families. BWA MEM version 0.7.17 was used to map the genome resequencing reads to genome. Mosdepth v0.3.3 was used to estimate per-base sequencing depth and sequencing depth. GATK version 4.2.6.1 was used to estimate the polymorphisms of ToEVEs. OrthoFinder version 2.5.4 was used to determine orthologous groups in gen evolving analysis. Bowtie2 v2.3.5.1 was used to investigate the transcript abundance of ToEVEs. Bowtie v1.2.3 was used to map the small RNA (sRNA) reads to transcripts. Mascot search engine v2.3.02 was utilized for searching potential ToEVE-encoded peptides in Laodelphax striatellus. MaxQuant v1.6.5.0 was utilized for searching potential ToEVE-encoded peptides in Nilaparvata lugens. sgRNAcas9 algorithm 3.0.5 was used to design sgRNA. HISAT2 v2.1.0 was used to align transcriptomic reads to the reference genome. SAMtools v1.7 was used to filter the low-quality alignments. Cufflink v2.2.1 was used to calculate transcripts per million expression values. The DEseq2 v2.2.1 was used to analyze the DEGs. TBtools v1.0697 was used to perform GO enrichment analyses. The ORF Finder server (https://www.ncbi.nlm.nih.gov/orffinder) was used to predicate open reading frames. The FASTX-Toolkit v0.0.14 was used to quality-control the raw reads of sRNAs. Primer Premier v6.0 was used to design qPCR primers. The codes used in mapping, depth estimation and polymorphism level analysis have been deposited in Github: https://github.com/lyy005/TotiEVEs.

For manuscripts utilizing custom algorithms or software that are central to the research but not yet described in published literature, software must be made available to editors and reviewers. We strongly encourage code deposition in a community repository (e.g. GitHub). See the Nature Portfolio guidelines for submitting code & software for further information.

Data

Policy information about availability of data

All manuscripts must include a data availability statement. This statement should provide the following information, where applicable:

- Accession codes, unique identifiers, or web links for publicly available datasets
- A description of any restrictions on data availability
- For clinical datasets or third party data, please ensure that the statement adheres to our policy

The RNA-seq data used for totivirus identification in Nilaparvata lugens and Sogatella furcifera have been deposited in the NCBI Sequence Read Archive (SRA) under accession number SRR19073262 [https://www.ncbi.nlm.nih.gov/sra/SRR19073262] and SRR11729951 [https://www.ncbi.nlm.nih.gov/sra/SRR11729951], respectively. Sequence data can be found in GenBank under the following accession numbers: Nilaparvata lugens toti-like virus 1, ON402804 [https://www.ncbi.nlm.nih.gov/nuccore/ON402804]; Sogatella furcifera toti-like virus 1, ON402805 [https://www.ncbi.nlm.nih.gov/nuccore/ON402805]; Sogatella furcifera toti-like virus 2, ON402806 [https://www.ncbi.nlm.nih.gov/nuccore/ON402806]; Fushun totivirus 2, MZ210014 [https://www.ncbi.nlm.nih.gov/nuccore/MZ210014]; Laodelphax striatellus iflavirus 1, MG815140.1 [https://www.ncbi.nlm.nih.gov/nuccore/MG815140.1]; Nilaparvata lugens honeydew virus-1, NC_038302.1 [https://www.ncbi.nlm.nih.gov/nuccore/NC_038302.1]; Nilaparvata lugens honeydew virus-2, NC_021566.1 [https://www.ncbi.nlm.nih.gov/nuccore/NC_021566.1]; Nilaparvata lugens honeydew virus-3, NC_021567.1 [https://www.ncbi.nlm.nih.gov/nuccore/NC_021567.1], Nilaparvata lugens reovirus, GCF_000852065.1 [https://www.ncbi.nlm.nih.gov/datasets/genome/GCF_000852065.1], Sogatella furcifera honeydew virus 1, MG818986.1 [https://www.ncbi.nlm.nih.gov/nuccore/MG818986.1], Sogatella furcifera totivirus 1, NC_040633.1 [https://www.ncbi.nlm.nih.gov/nuccore/NC_040633.1], and Sogatella furcifera totivirus 2, NC_040704.1 [https://www.ncbi.nlm.nih.gov/nuccore/NC_040704.1]; piggyBac transposable element-derived protein 3-like, XP_039275920.1 [https://www.ncbi.nlm.nih.gov/protein/XP_039275920.1]. The sRNA-seq raw data of N. lugens, Laodelphax striatellus and S. furcifera can be found in NCBI SRA under accession number: PRJNA834899 [https://www.ncbi.nlm.nih.gov/sra/?term=PRJNA834899], PRJNA834958 [https://www.ncbi.nlm.nih.gov/sra/?term=PRJNA834958], and PRJNA834900 [https://www.ncbi.nlm.nih.gov/sra/?term=PRJNA834900], respectively. The RNA-seq raw data used for the analysis of DEGs between N. lugens strains of WT and KO-M1 can be found in NCBI SRA under accession number PRJNA987576 [https://www.ncbi.nlm.nih.gov/bioproject/PRJNA987576]. The genomes of N. lugens, L. striatellus, and S. furcifera, were retrieved from NCBI genome database with the accession numbers GCA_014356525 [https://www.ncbi.nlm.nih.gov/datasets/genome/GCF_014356525.2], GCA_014465815 [https://www.ncbi.nlm.nih.gov/datasets/genome/GCA_014465815.1], and GCA_014356515.1 [https://www.ncbi.nlm.nih.gov/datasets/genome/GCA_014356515.1], respectively. The LC-MS/MS-based proteomic data are available in ProteomeXchange under the accession numbers: PXD023965 [https://proteomecentral.proteomexchange.org/cgi/GetDataset?ID=PXD023965], PXD036431 [https://proteomecentral.proteomexchange.org/cgi/GetDataset?ID=PXD036431], PXD043983 [https://proteomecentral.proteomexchange.org/cgi/GetDataset?ID=PXD043983], and PXD044065 [https://proteomecentral.proteomexchange.org/cgi/GetDataset?ID=PXD044065]. The protein data of three planthoppers can be found in InsectBase 2.0 under the following ID: N. lugens, IBG_00572 [http://v2.insect-genome.com/Organism/572], L. striatellus, IBG_00477 [http://v2.insect-genome.com/Organism/477] and S. furcifera, IBG_00709 [http://v2.insect-genome.com/Organism/709]. The NCBI SRA accession numbers for the transcriptome raw data used in the expression analysis of planthoppers and representative arthropods are provided in the Supplemental Table 3 and Supplemental Table 5, respectively. The NCBI genome accessions for the representative arthropods are provided in the Supplemental Table 4. The authors declare that the data supporting the findings of this study are available in the paper, Supplemental Table 1-6, and Supplemental Data 1-2. Source data are provided with this paper.

Research involving human participants, their data, or biological material

Policy information about studies with human participants or human data. See also policy information about sex, gender (identity/presentation), and sexual orientation and race, ethnicity and racism.

Reporting on sex and gender	not applicable
Reporting on race, ethnicity, or other socially relevant groupings	not applicable
Population characteristics	not applicable
Recruitment	not applicable
Ethics oversight	not applicable

Note that full information on the approval of the study protocol must also be provided in the manuscript.

Field-specific reporting

Please select the one below that is the best fit for your research. If you are not sure, read the appropriate sections before making your selection.

☒ Life sciences ☐ Behavioural & social sciences ☐ Ecological, evolutionary & environmental sciences

For a reference copy of the document with all sections, see nature.com/documents/nr-reporting-summary-flat.pdf

Life sciences study design

All studies must disclose on these points even when the disclosure is negative.

Sample size	No statistic method was used to pre-determine the sample size. Sample sizes were chosen based on established protocols and previous publications. Generally three independent biological replicates were done for each experiment. The insect bioassays (survival, developmental

	duration, adult longevity, female-male ratio, fecundity) were referred to Huang et al. Nature Communications, 2023 (10.1038/s41467-023-36403-5) and Xue et al., PLoS Genetics, 2021 (10.1371/journal.pgen.1009653).
Data exclusions	No data were excluded from the analyses, except for the sterile female in the fecundity analyses
Replication	All attempts at replication were successful. Generally, experiments were repeated three times. For CRISPR/Cas9-mediated knockout analyses, two independent insect lines were performed.
Randomization	All samples were allocated randomly into experimental groups.
Blinding	All investigation were blinded to group allocation during data collection and analysis

Reporting for specific materials, systems and methods

We require information from authors about some types of materials, experimental systems and methods used in many studies. Here, indicate whether each material, system or method listed is relevant to your study. If you are not sure if a list item applies to your research, read the appropriate section before selecting a response.

Materials & experimental systems

n/a	Involved in the study
☐	☒ Antibodies
☐	☒ Eukaryotic cell lines
☒	☐ Palaeontology and archaeology
☐	☒ Animals and other organisms
☒	☐ Clinical data
☒	☐ Dual use research of concern
☒	☐ Plants

Methods

n/a	Involved in the study
☒	☐ ChIP-seq
☒	☐ Flow cytometry
☒	☐ MRI-based neuroimaging

Antibodies

Antibodies used	1. anti-NlToEVE14 serum (1:5,000 dilution, WB) was prepared by immunizing rabbits with purified His-NlToEVE14 (260-529aa) proteins, followed by injecting Rabbit via the custom service of Huaan Biotechnology Company (Hangzhou, China).<br>2. His-tag antibodies (1:10,000 dilution, WB). REF: # MA1-21315; Vendor website: https://www.thermofisher.cn/cn/zh/antibody/product/6x-His-Tag-Antibody-clone-HIS-H8-Monoclonal/MA1-21315.<br>3. Flag-tag antibodies (1:10,000 dilution, WB).REF: # MA1-91878; Vendor website: https://www.thermofisher.cn/cn/zh/antibody/product/DYKDDDDK-Tag-Antibody-clone-FG4R-Monoclonal/MA1-91878.<br>4. Horseradish peroxidase (HRP)-conjugated goat anti-rabbit IgG antibody (1:10,000 dilution, WB), REF: # 31460; Vendor website: https://www.thermofisher.com/antibody/product/Goat-anti-Rabbit IgG H L Secondary-Antibody-Polyclonal/31460.<br>5. Horseradish peroxidase (HRP)-conjugated goat anti-mouse IgG antibody (1:10,000 dilution, WB), REF: # 31430; Vendor website: https://www.thermofisher.com/antibody/product/Goat-anti-Mouse IgG H L Secondary-Antibody-Polyclonal/31430
Validation	1. We validate the specificity of anti-NlToEVE14 serum by comparing the dsGFP-treated insect against the dsNIETLVE14-treated, as well as comparing wild-type (WT) and NlToEVE14 knockout mutants. The results were provided in Figure 4c and Supplementary Figure 6.<br>2. The commercial antibodies were validated to be useful by manufacturer.

Eukaryotic cell lines

Policy information about cell lines and Sex and Gender in Research

Cell line source(s)	HEK293T cells used in this study were obtained from ATCC CRL-3216.
Authentication	HEK293T cells was authenticated by morphology in our lab.
Mycoplasma contamination	The cell line was not tested for Mycoplasma contamination.
Commonly misidentified lines<br>(See ICLAC register)	No commonly misidentified cell lines were used in the study.

Animals and other research organisms

Policy information about studies involving animals; ARRIVE guidelines recommended for reporting animal research, and Sex and Gender in Research

Laboratory animals	The Nilaparvata lugens used in this study has been inbred following a sib-sib mating protocol for 13 generations, and reared in our lab for more than 100 generations. The Laodelphax striatellus and Sogatella furcifera have been inbred following a sib-sib mating

protocol for 5 generations, and reared in our lab for more than 10 generations. It is difficult to distinguish male and female at nymph stages of three planthoppers. Therefore, we randomly select the male and female nymphs for analysis.
For the tissue assay, salivary gland (50), gut (20), fat body (20), leg (10), carcass (10), and wing buds(10) were collected from 5th instar N. lugens, respectively. The number of insects in each sample was given in the parentheses above.
For the developmental assay, 3rd and 4th instar nymphs were reared on rice seedlings and used to obtain the newly emerged 4th and 5th instar nymphs, respectively. The newly emerged nymphs were then further maintained in a climate chamber and collected at every 12 hour (4th instar nymph) and 24 hour (5th instar nymph) intervals, respectively.
For survival and developmental duration assay, newly hatched 1st instar nymphs were individually reared on 4-5-leaf stage rice seedlings, and the survival rates and stage durations were recorded every 12 h. In total, 50-60 replicates were performed for each strains.
For longevity analysis, newly emerged male or female adults were individually reared on 4-5-leaf stage rice seedlings, and the death time was recorded every day. In total, 50-60 replicates were performed for each mutant strains.
For fecundity analysis, the newly emerged adults were paired and allowed to oviposit for 10 days. The number of hatched offspring and dead embryos was counted. In total, 15-20 replicates were performed for each strain.

Wild animals

This study did not involve wild animals.

Reporting on sex

It is difficult to distinguish male and female at nymph stages of three planthoppers. Therefore, we randomly select the male and female nymphs for analysis.

Field-collected samples

No field collected samples were used in the study.

Ethics oversight

We only use planthopper species for the experiments in this study. No ethical approval or guidance was required

Note that full information on the approval of the study protocol must also be provided in the manuscript.

Plants

Seed stocks

Not applicable.

Novel plant genotypes

Not applicable.

Authentication

Not applicable.
